# Genome Sequencing and Analysis of a Type A *Clostridium perfringens* Isolate from a Case of Bovine Clostridial Abomasitis

**DOI:** 10.1371/journal.pone.0032271

**Published:** 2012-03-08

**Authors:** Victoria J. Nowell, Andrew M. Kropinski, J. Glenn Songer, Janet I. MacInnes, Valeria R. Parreira, John F. Prescott

**Affiliations:** 1 Department of Pathobiology, University of Guelph, Guelph, Ontario, Canada; 2 Laboratory for Foodborne Zoonoses, Public Health Agency of Canada, Guelph, Ontario, Canada; 3 Department of Molecular and Cellular Biology, University of Guelph, Guelph, Ontario, Canada; 4 Department of Veterinary Microbiology and Preventive Medicine, Iowa State University, Ames, Iowa, United States of America; University of Edinburgh, United Kingdom

## Abstract

*Clostridium perfringens* is a common inhabitant of the avian and mammalian gastrointestinal tracts and can behave commensally or pathogenically. Some enteric diseases caused by type A *C. perfringens*, including bovine clostridial abomasitis, remain poorly understood. To investigate the potential basis of virulence in strains causing this disease, we sequenced the genome of a type A *C. perfringens* isolate (strain F262) from a case of bovine clostridial abomasitis. The ∼3.34 Mbp chromosome of *C. perfringens* F262 is predicted to contain 3163 protein-coding genes, 76 tRNA genes, and an integrated plasmid sequence, Cfrag (∼18 kb). In addition, sequences of two complete circular plasmids, pF262C (4.8 kb) and pF262D (9.1 kb), and two incomplete plasmid fragments, pF262A (48.5 kb) and pF262B (50.0 kb), were identified. Comparison of the chromosome sequence of *C. perfringens* F262 to complete *C. perfringens* chromosomes, plasmids and phages revealed 261 unique genes. No novel toxin genes related to previously described clostridial toxins were identified: 60% of the 261 unique genes were hypothetical proteins. There was a two base pair deletion in *virS*, a gene reported to encode the main sensor kinase involved in virulence gene activation. Despite this frameshift mutation, *C. perfringens* F262 expressed perfringolysin O, alpha-toxin and the beta2-toxin, suggesting that another regulation system might contribute to the pathogenicity of this strain. Two complete plasmids, pF262C (4.8 kb) and pF262D (9.1 kb), unique to this strain of *C. perfringens* were identified.

## Introduction

*Clostridium perfringens* is a Gram-positive bacterium that flourishes in the nutrient-rich environment of the mammalian and avian gastrointestinal tract and causes disease when relevant toxins are expressed [1]. While *C. perfringens* is usually a harmless member of the normal microflora, under certain conditions it can multiply rapidly and secrete toxins and degradative enzymes that are associated with serious enteric disease. Clostridial abomasitis is a severe enteric disease of calves that often presents as fatal hemorrhagic enteritis involving the abomasum and upper small intestine [2]. The disease is thought to be caused by type A *C. perfringens*, though the mechanisms of pathogenesis are not understood. Calves display acute necrotizing hemorrhagic inflammation, with emphysema in the abomasum and often in the upper small intestine [2], [3], and present clinically with abomasal tympany, abdominal bloating and distension [4]. The disease is acute and death often occurs soon after the onset of clinical signs. Predisposing host factors are known to include dietary changes [3], [5], but the contributing pathogen factors are poorly understood. Although the discovery of toxins has aided in the understanding of some other type A-mediated enteric diseases, such as the recent recognition of the critically important pore-forming toxin NetB in isolates causing necrotic enteritis in chickens [6], no known toxins have been convincingly or reproducibly linked to clostridial abomasitis. The suggestion has been made that the beta2-toxin may be involved, but experimental findings are sparse and often contradictory [7]–[11].

We hypothesized that isolates of type A *C. perfringens* associated with bovine clostridial abomasitis produce one or more novel toxins related to those of other clostridia causing severe enteric disease. More specifically, we hypothesized that these isolates would possess a pore-forming toxin related to the beta-toxin [12], the NetB toxin [6], or the beta2-toxin [13]. We also hypothesized that, typical of *C. perfringens* isolates causing severe enteric disease in animals [14]–[16], this toxin gene would be present on a plasmid. To identify genetic factors potentially involved in bovine clostridial abomasitis, the sequence of the genome of a type A isolate of *C. perfringens* from a case of the disease was sequenced. Unique areas of the F262 genome were identified by comparison to the complete *C. perfringens* sequences: Strain 13 [17], ATCC 13124 and SM101 [18]. Complete plasmid and phage sequence were also included in the analysis. Identification of genomic regions unique to *C. perfringens* F262 should help to identify important virulence-associated genes and to add to our understanding of the pan-genome of virulent *C. perfringens* [18].

## Results

### Bacterial isolates

Isolate F262 was recovered in large numbers from the abomasal content of a calf that died of clostridial abomasitis, diagnosed on the basis of typical gross and histopathological lesions of acute necrotizing inflammation, with emphysema. It was identified as a type A, *cpb2*-positive *C. perfringens* by the Animal Health Laboratory (University of Guelph, Guelph, ON). The *cpb2* gene was later identified through genome sequencing as the atypical variant. Pulsed-field gel electrophoresis showed that a *C. perfringens* isolate from another calf in the clostridial abomasitis outbreak shared the same pulsed-field gel electrophoresis restriction pattern as *C. perfringens* F262 (data not shown).

### Whole-genome sequencing, assembly and annotation

**Figure 1:**
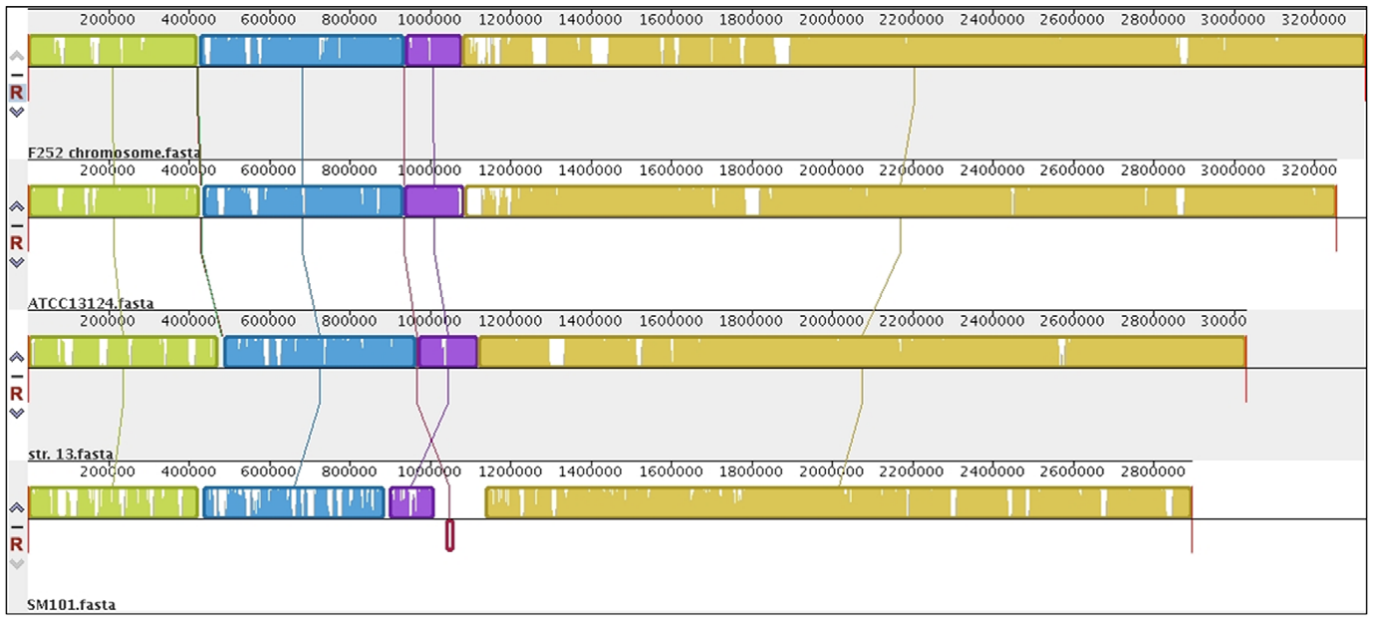
ProgressiveMauve alignment of *Clostridium perfringens* F262 chromosome versus the chromosomes ATCC13124, Strain 13 and SM101.

The whole-genome sequencing generated a total of 757,481 reads, with 95.6% of the reads assembled into 60 contigs of varying sizes. Coverage was estimated at 80-fold. The 60 contigs totaling, 3,464,572 bp (3.46 Mbp), had an average contig size of 57,742 bp. Twenty-five of the 60 contigs could be positioned on the NcoI optical map (Figure S1). The optical map was approximately 3.34 Mbp in size. Since the 25 placed contigs represented 3.1 Mbp in total, ∼93% of the chromosome was thus assembled using the optical mapping alone. By comparing the BLASTX results of the 5′ and 3′ ends of the mapped and unmapped contigs, the positions of a further 17 unmapped contigs were predicted and positioned within the pseudochromosome. The revised 3.33 Mbp pseudochromosome was compared to the three complete *C. perfringens* chromosomes available in NCBI in order to assess the validity of the assembly (Figure 1). This comparison showed that the chromosome of *C. perfringens* F262 is similar to that of Strain 13, ATCC 13124 and SM101. The F262 pseudochromosome was slightly larger than the other three chromosomes and contained unique regions, but there were no major rearrangements. An integrated plasmid sequence (∼18 kb) was found and linked by PCR to neighbouring contigs to create “Cfrag”. Two plasmid fragments, pF262A (48.5 kb) and pF262B (50.0 kb), were also created by linking smaller contigs but could not be circularized by PCR (details not shown). Two complete plasmids, pF262C (4.8 kb) and pF262D (9.1 kb), were identified and shown to be circular by PCR amplification. A total of 3244 genes, 3163 protein-coding genes, 76 tRNA genes and 5 rRNA genes were identified using the Prokaryotic Genomes Automatic Annotation Pipeline (GenBank accession number: AFES00000000). From this analysis, it was also possible to demonstrate that *C. perfringens* F262 has a number of virulence-related genes first described by Shimizu *et al*. (2002) (Table S1) [17].

### Identification of unique nucleotide sequence using PanSeq

**Figure 2:**
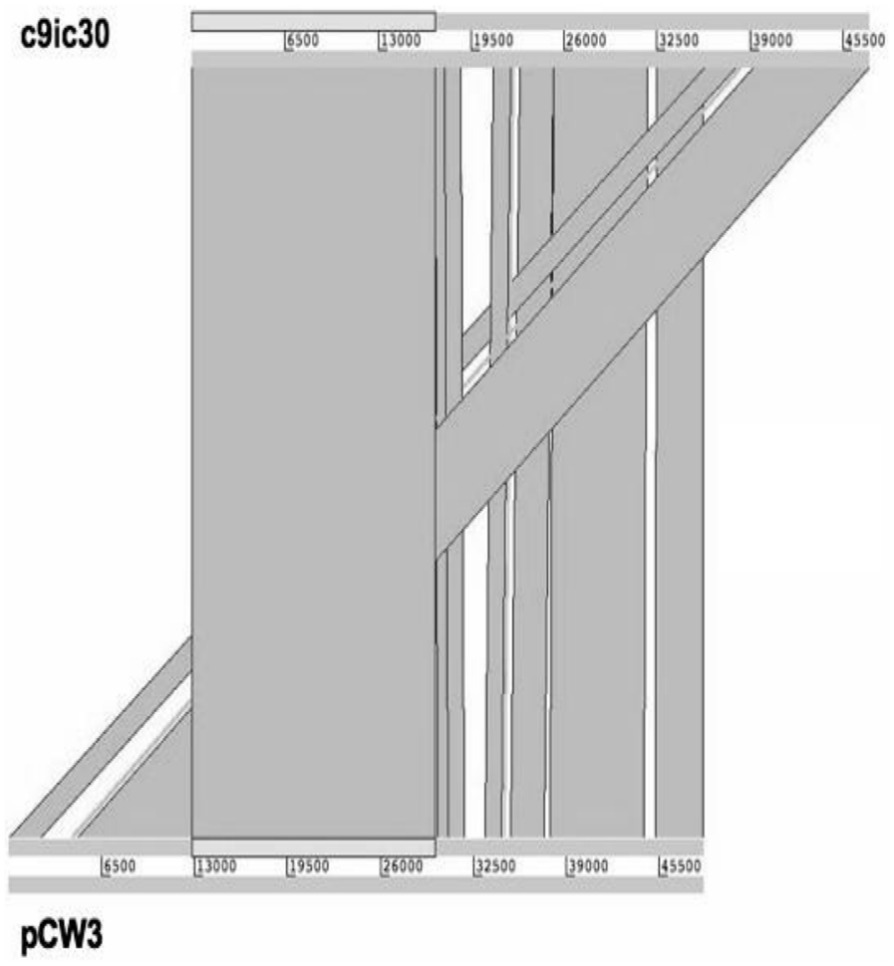
WebACT comparison of pF262A to pCW3.

**Figure 3:**
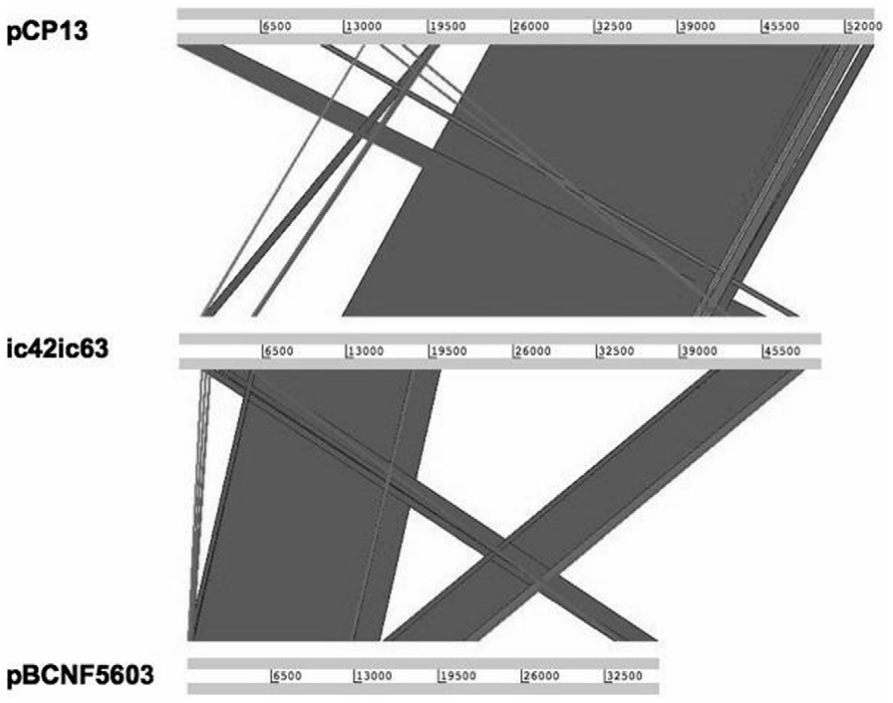
WebACT comparison of pF262B to pCP13 and pBCNF5603.

Using PanSeq [19], ∼300 kb containing 261 ORFs were identified in *C. perfringens* F262 that were absent in Strain13, ATCC 13124, SM101 and from all complete *C. perfringens* plasmid and phage sequences available in GenBank at the time of analysis. When the unique regions in the *C. perfringens* F262 chromosome were compared to all complete *C. perfringens* plasmid and phage sequences, only two of the ORFs in Contig_14 and one ORF in contig00062 were found not to be unique (Cfrag was not included in this analysis). A summary of the unique ORFs found by PanSeq can be found in Table 1, Cfrag results in Table S2, and all other PanSeq results found in Tables S3 and S4. Large unique regions were found in Contig_14 (53 ORFs) and Contig_6 (32 ORFs). Sequences unique to *C. perfringens* F262 included site-specific recombinases (HA1_01897, HA1_06197, HA1_00723, HA1_07162), unique phage-like proteins (HA1_08482, many in Contig_14, HA1_08347, HA1_13167, HA1_13212, HA1_13597), unique sigma factors (HA1_06057, HA1_13097) and unique predicted transcriptional regulators (HA1_06282, HA1_13192, HA1_13197, HA1_07182). The two large plasmid-associated fragments, pF262A and pF262B, contained no and three unique ORFs, respectively. One hundred and sixty-two of the 261 unique ORFs (62%) identified by PanSeq were hypothetical proteins (Table 1). Although Cfrag, pF262C and pF262D contained plasmid-related genes, none were identical to *C. perfringens* plasmid sequences in GenBank from completely sequenced and circularized plasmids, but did display similarity to sequences from incomplete genomes available in GenBank. Plasmid-related fragments pF262A and pF262B compared to complete *C. perfringens* plasmid sequences using WebACT [20] showed that pF262A was very similar to pCW3 with few differences, explaining why after PanSeq comparison to the *C. perfringens* plasmid sequences nothing remained unique (Figure 2). The ends of pF262B were similar to pBCNF5603 (NC_006872), and the middle region was most similar to pCP13 (NC_003042) (Figure 3).

**Table 1:** List of the contigs with unique open reading frames (ORFs) identified by PanSeq after comparison to all *Clostridium perfringens* sequences (chromosome, phage and plasmid).

Contig	Total CDS	Total unique CDS	#Hypothetical proteins	#Regulatory proteins	#Phage-related proteins	#Recombination-associated proteins
pF262A	56	0	-	-	-	-
pF262B	46	3	3	-	-	-
pF262C	7	7	5	1	-	-
pF262D	13	13	8	1	-	-
Cfrag	237	68	36	5	-	2
Contig_1	337	11	7	1	-	-
Contig_10	74	12	8	1	-	1
Contig_12	106	19	16	1	2	-
Contig_14	119	53	31	3	15	1
Contig_15	83	5	4	-	-	1
Contig_28	35	4	2	-	1	-
Contig_4	161	8	1	-	-	-
Contig_6	194	32	25	3	3	-
Contig_8	98	7	-	-	-	-
Contig_9	147	13	11	1	-	1
contig00062	18	6	5	-	-	-
**TOTAL**	1731	261	162	17	21	6

### VirS frameshift mutation

**Figure 4:**
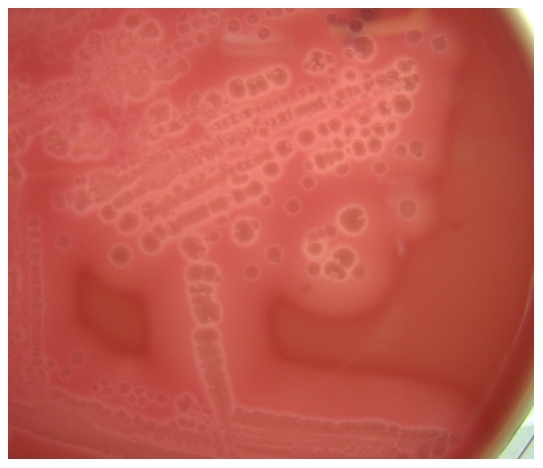
F262 on blood agar shows the double hemolysis typical of perfringolysin O and alpha-toxin.

Sequencing of a PCR amplicon confirmed the presence of a 2 bp deletion in the *C. perfringens* F262 VirS gene first detected in the original sequencing data. As elaborated in the discussion, this 2 bp deletion causes a downstream stop codon to become in frame. To assess the production of alpha-toxin and perfringolysin O, two toxins controlled by the VirR/VirS system, *C. perfringens* F262 was plated on blood agar (Figure 4). *Clostridium perfringens* F262 retained the ability to produce a double zone of hemolysis on blood agar, consistent with the notion that alpha-toxin and perfringolysin O were still being expressed.

**Figure 5:**
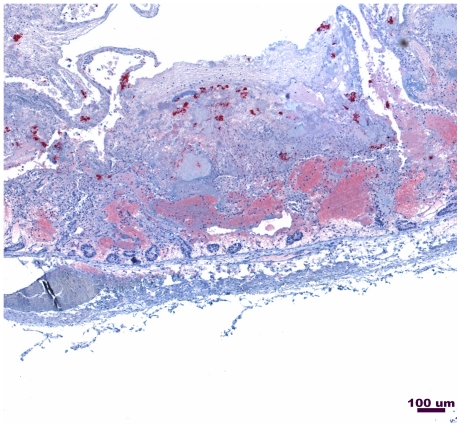
Immunohistochemical staining for beta2-toxin of intestinal tissue cross-section from Calf F262.

**Figure 6:**
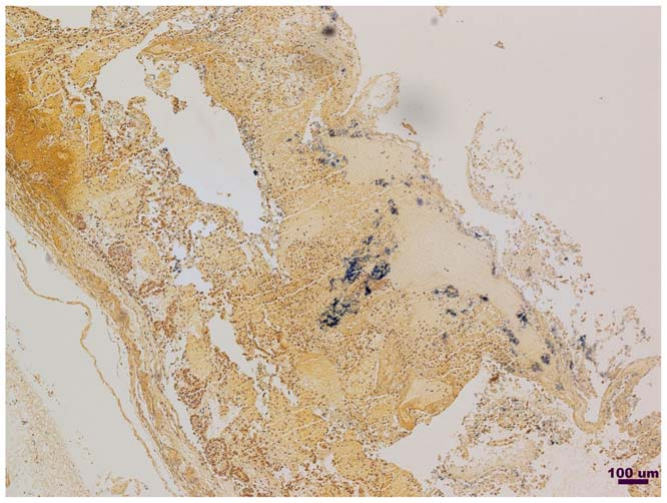
Tissue Gram stain of a formalin-fixed intestinal tissue cross-section from Calf F262.

To assess the expression of the beta2-toxin, another toxin controlled by the VirR/VirS system, immunohistochemistry using rabbit polyclonal antiserum to recombinant consensus beta2-toxin was performed on a formalin-fixed histological section of small intestinal lesion from the F262 calf (Figure 5). Staining revealed the production of beta2 toxin and the presence of Gram-positive rods typical of *C. perfringens* in the same tissue sample (Figure 6).

## Discussion

This study is the fourth description of a *C. perfringens* genome and the first detailed examination of an isolate from a case of bovine clostridial abomasitis. Using whole-genome sequencing to understand the possible basis of virulence is more rapid than “traditional” approaches, although it did not permit the identification of truly novel virulence genes or regulatory differences that affect the expression of known virulence genes. This study has advanced understanding of the pathogenesis of clostridial abomasitis by suggesting that the disease is not associated with a toxin variant of known clostridial toxins, such as the beta-toxin [6], as originally hypothesized. This study also suggests that this disease may rather involve one of the many hypothetical proteins or other ORFs identified as unique to this isolate, or more simply known toxins such as the alpha-toxin and perfringolysin O. These major conclusions are discussed further below.

### F262 chromosome

The approximate size of the F262 chromosome is 3.34 Mbp based on optical mapping. This is slightly larger than the other three complete *C. perfringens* chromosomes reported to date [18]. The unique regions identified by PanSeq were confined to 15 contigs/supercontigs and unique ORFs were often clustered closely together, consistent with other *C. perfringens* chromosomal comparisons [18].

The presence of unique transcriptional regulator and sigma factor homologs suggests that the regulation of transcription may have elements unique to *C. perfringens* F262; determining whether this extends to the regulation of virulence genes would be worthwhile. Extensive phage-related sequences, mostly with low sequence similarity to phiSM101, a phage in *C. perfringens* SM101 [21], are present in Contig_14. Phage-related sequence has also been identified in the chromosomes of *C. perfringens* strains, ATCC13124, SM101 and Strain 13 [18]. A DNA adenine methylase similar to one found in phiSM101 was also found on contig00062 upstream of a unique ORF annotated as a putative N-acetylmuramoyl-L-alanine amidase (Table S3), described as an “endolysin” in JGS1987, a type E *C. perfringens* isolate from bovine enteritis. The presence of such phage-derived endolysins has been suggested to have potential applications in biocontrol of *C. perfringens* [21]. Although temperate phages have been associated with virulence in other bacterial species, there is no evidence for their involvement in the virulence of *C. perfringens*.

### Chromosomally integrated plasmid sequence, Cfrag

To date, *cpe* (enterotoxin) is the only virulence gene that has been shown to move between plasmid and the chromosome in *C. perfringens* [22]–[24], although integration of plasmid DNA has been demonstrated in other bacterial species [25], [26]. Cfrag (Table S2) contains an ORF similar to the collagen adhesin-encoding gene, *cna*, first identified in pCP13 by Shimizu *et al*. (2002), and suggested to facilitate colonization. Interestingly, two other *cna* homologs are present in *C. perfringens* F262 [17]. It is tempting to speculate that, if expressed, these genes might be associated with enhanced intestinal colonization in the initial stages of infection, but this remains to be determined experimentally. The presence of *cna* was the first indication that the *C. perfringens* F262 chromosome contained plasmid DNA. The middle section of Cfrag contained a number of other ORFs that had minor sequence similarity to pCP13. This finding suggests that the ∼18 kb of plasmid-related, but chromosomally-integrated, sequence is likely distantly related to pCP13 and adds to the still incomplete picture of *C. perfringens* plasmid evolution [14], [16], [27].

One ORF of interest in the Cfrag contig, HA1_04912 (Table S2), contains a domain sharing homology with a caspase domain found in *Faecalibacterium prausnitzii* L2-6. Although this bacterium is typically associated with anti-inflammatory properties [28], caspases participate in cellular cascades that release pro-inflammatory cytokines and regulate apoptosis [29]. Such cascades are critical during microbial infections. Intense inflammation is a feature of bovine clostridial abomasitis and it would be of interest to search for this caspase-related gene in isolates from other cases.

Though not apparently related to virulence, a CRISPR element was identified in Cfrag. Clustered Regularly Interspaced Short Palindromic Repeat (CRISPR) elements are found frequently in bacteria. It is suspected that the main function of the CRISPR/Cas system is to protect against the introduction of foreign DNA, particularly phages, but also plasmids [30]. It will be of interest to determine whether the presence of CRISPR systems is typical of bovine clostridial abomasitis isolates. Interestingly, there is emerging evidence that CRISPR systems may have functions other than simply defense against foreign DNA. For example, CRISPR systems have been shown to be involved in inhibiting phage-associated biofilm formation in *Pseudomonas aeruginosa* [31]. Despite the presence of this CRISPR element, F262 had one large bacteriophage cluster elsewhere in the genome.

### Plasmid-related fragments

The 48,504 bp pF262A plasmid fragment was created by linking two smaller contigs. Southern blotting and pulsed-field analysis indicated that pF262A was ∼55 kb (data not shown), but the missing piece has not been successfully identified. Plasmid pF262A is almost identical to pCW3 (NC_010937) (Figure 2), a 47 kb conjugative tetracycline resistance plasmid [32], except for a 2 kb fragment found on pF262A that contains the beta2-toxin gene. As in pCW3, the *tcp* conjugative locus is present in a Tn*916*-like region [32]. Future studies will be needed to identify the missing 6–9 kb piece of pF262A.

The 50,048 bp plasmid fragment pF262B is the second large plasmid piece in *C. perfringens* F262, but, unlike pF262A, pF262B does not appear to have a conjugative locus. Several attempts to join the ends using long-range PCR were unsuccessful (details not shown). The fragment pF262B is most similar to plasmids pCP13 and pBCNF5603 (Figure 3), suggesting that pF262B was formed by a recombination event between two *tcp*-negative plasmids related to pCP13 and pBCNF5603. Only three ORFs were unique to pF262B when compared to other complete *C. perfringens* sequences and were all hypothetical proteins (HA1_15670, HA1_15905 and HA1_15910). Interesting features of pF262B include a two-component system as well as a putative ABC transport system also found on pBCNF5603. In addition, pF262B contained homologs of VirB4 and VirD4 components of a type IV secretion system (T4SS) similar to hypothetical proteins found in pCP13. VirB4 is part of the T4SS transmembrane core and VirD4 the substrate receptor [33]. VirB4 and VirD4 homologs are also present in the Cfrag supercontig. It is likely that these T4SS-related components in *C. perfringens* F262 are part of the systems used for exchanging the DNA of integrative and conjugative mobile elements [34].

Our original hypothesis was that F262 would possess a plasmid-encoded novel toxin gene, possibly related to the beta toxin, similar to the recent discovery of *netB* by Keyburn and others [6]. It is possible but seems unlikely that any novel toxin genes were missed, because of both the extensive coverage and of the presence of only single conjugative plasmid typical of *C. perfringens* plasmids, so that there were unlikely to be extensive repetitive DNA sequences that interfered with sequencing. Despite extensive efforts, the two large plasmids could not be closed by PCR nor the unplaced plasmid fragments placed in either of the plasmids.

pF262C (4,809 bp) and pF262D (9,136 bp) are two small plasmids that are not similar to any other complete *C. perfringens* plasmid sequences reported to date. Plasmid pF262C contained a replication protein (HA1_15992) similar to the Rep1 protein in strain JGS1495, a type C *C. perfringens* isolate from a pig with diarrhea. The other seven ORFs in pF262C were hypothetical proteins with low similarity to genes in a variety of other species, including HA1_16002, a unique predicted transcriptional regulator not previously identified in *C. perfringens*. Plasmid pF262D sequences were most similar to sequences in *C. perfringens* strains JGS1721 (type D from sheep enteritis) and JGS1987 (type E from cow enteritis). The replication protein was most similar to Rep in JGS1721. Plasmid pF262D also contained two predicted recombination-related elements and one transposase, suggesting it may be a hot spot for recombination. Because both of these plasmids are most similar to sequences from enteritis-related isolates, it is possible that they contribute to virulence. It will be worthwhile to search for these plasmids in other hemorrhagic abomasitis isolates.

Virulence-related genes first described by Shimizu *et al*. (2002) were identified in *C. perfringens* F262 and may contribute to virulence (Table S1) [17]. The shared ORFs include a number of hemolysins, sialidases and hyaluronidases. *C. perfringens* F262 also contains all three sialidases present in ATCC 13124: NanI (HA1_03714), NanJ (HA1_02772) and NanH (HA1_04722). Further work will be required to determine the contribution of virulence determinants such as alpha-toxin and perfringolysin O, known to be important in the virulence of some *C. perfringens* diseases [35], to bovine clostridial abomasitis. One important finding in this work, discussed below, was the frameshift mutation in VirS, which unpredictably allowed expression of VirR-controlled toxin genes.

### VirS frameshift mutation

In Gram-positive bacteria (including *C. perfringens*) there are two-component regulatory systems that contain a transmembrane sensor kinase that samples the extracellular environment for auto-inducing peptides (AIPs) [36]. Such a system in *C. perfringens* has recently been described and compared to the well-known *agr* quorum-sensing cascade in *Staphylococcus aureus* [37], [38]. In *C. perfringens*, VirS relays information to a cytoplasmic response regulator (VirR) through autophosphorylation of the C-terminal domain. Once phosphorylated, the response regulator can bind to the promoter region of genes and activate or suppress transcription. In *C. perfringens*, it is postulated that phosphorylation occurs at H255 of VirR when the C-terminal end of VirS catalyzes the transfers of a phosphate from ATP [39]. Cheung *et al*. (2009) have shown that a tyrosine substitution at the C335 position reduces autophosphorylation and that substitution of G402D prevents it entirely [40].

The 2 bp deletion in *C. perfringens* F262 changes amino acid 335 from a cysteine to a tyrosine, in a remarkable coincidence identical to the substitution in the earlier experimental study [40]. This frameshift introduces a stop codon at amino acid 339, truncating just more than 100 amino acids at the C-terminus, which includes the domains that bind and position ATP. As shown experimentally, this should prevent autophosphorylation of VirS and disrupt the phosphorelay. Without this cascade, VirR should be incapable of transcriptional activation of the VirR regulon. Virulence genes activated indirectly or directly by VirR include *cpb2* (beta2-toxin), *cna* (collagen adhesin), *cpa* (alpha-toxin), *pfoA* (perfringolysin O), and *ccp* (clostripain) [41], [42]. Despite the frameshift mutation shown by Cheung *et al*., (2009) to prevent phosphorylation of VirR, *C. perfringens* F262 secreted perfringolysin O and alpha-toxin (Figure 4) [40]. In addition, immunohistochemistry revealed that atypical beta2-toxin was produced *in vivo* (Figure 5).

The ability to produce these toxins despite an apparently non-functional VirR/VirS system has not been previously reported in *C. perfringens* and is unusual since the VirR/VirS system is central to the control of virulence and related genes [41]–[43]. The two most likely reasons for the continued expression of these genes are either that another sensor kinase is phosphorylating VirR or that another transcriptional activator is involved. To date only two other transcriptional regulators, RevR [44] and CPE1446-CPE1447 [45], have been shown to regulate virulence in *C. perfringens*, so the former speculation seems more likely. The F262 chromosome contains a RevR homolog (HA1_03239 in Contig_1) as well as a CPE1446-CPE1447 homolog (HA1_09041/HA1_09046 in Contig_19) (Table S3). There are other putative sensor kinases within the *C. perfringens* F262 chromosome that may interact with VirR, most of which are shared with other complete *C. perfringens* genomes, but a unique sensor kinase (HA1_16132) was identified in an orphan contig (Table S4) and shares significant similarity to a sensor kinase found in JGS1721, a type D *C. perfringens* isolate from sheep enteritis. Future studies are needed to determine whether VirR is phosphorylated in *C. perfringens* F262 and whether inactivation of VirR disrupts hemolysis. If other genes within the VirR regulon are also constitutively expressed, this may be a contributor to virulence in the same manner that virulent gas gangrene strains often show increased expression levels of alpha-toxin [35]. It will also be important to determine if a truncated VirS protein is a characteristic of all bovine clostridial abomasitis isolates.

This is the first description of the genome sequence of a type A *C. perfringens* strain isolated from a case of bovine clostridial abomasitis. Although the findings of this study are not sufficient to explain or predict virulence, they add to the understanding of genome diversity in *C. perfringens* and contribute to our understanding of the pathogenesis of an important disease of calves. The discovery of an integrated plasmid sequence and of the frameshift mutation in *virS* is significant and should be further investigated. A clearer picture of virulence may emerge as more *C. perfringens* sequences become available and when experiments are done to elucidate the role of the many hypothetical proteins. Until then, future work should seek to better understand how regulation is involved in virulence and the attempt to find a “virulence signature” that predicts the virulence of type A bovine clostridial abomasitis isolates should continue.

## Materials and Methods

### Bacterial isolates

Two dead, 1-day-old Holstein Friesian calves were received by the Animal Health Laboratory at the University of Guelph, (Ontario, Canada) from a small outbreak of hemorrhagic clostridial abomasitis. The pathological diagnosis was clostridial abomasitis based on the extensive acute necrotizing hemorrhagic inflammation with emphysema in the abomasum and upper small intestine of the calf. *Clostridium perfringens* was isolated in large numbers from the abomasum and small intestine and identified as type A and atypical *cpb2*-positive by PCR based genotyping (data not shown). Pulsed-field gel electrophoresis was performed on *C. perfringens* F262 and another *C. perfringens* isolate from the same outbreak as previously described [46].

### Whole-genome sequencing, assembly and annotation

Whole genomic DNA was extracted using a modified version of the Qiagen bacterial DNA isolation protocol (Qiagen, Toronto, ON). Whole-genome sequencing was carried out by the McGill University and Genome Quebec Innovation Centre (Montreal, QC, Canada). A Titanium half sequence run (70–80× coverage) was performed using the Roche 454 GS FLX Titanium massively-parallel shotgun sequencing technology. Newbler v.2.3 software was used to assemble the raw reads into contigs. A culture of *C. perfringens* F262 was sent to OpGen® MapIt Services (Gaithersburg, MD) for optical mapping using the Argus™ optical mapping system. The optical map was analyzed using MapSolver™ software (OpGen, Gaithersburg, MD). Alignment against the optical map and additional manual placement based on BLASTX predictions were used to assemble a pseudochromosome. The pseudochromosome was compared to the three available complete *C. perfringens* chromosomes Strain 13 (GenBank Accession number NC 003366.1), ATCC 13124 (GenBank Accession number NC 008261.1) and SM101 (GenBank Accession number NC 008262.1) using progressive Mauve alignment software [47] in order to assess the accuracy of the assembly. Assembly of an integrated plasmid sequence (Cfrag) and plasmid fragments (pF262A, pF262B, pF262C and pF262D) was completed based on Southern blotting and PCR data. Plasmid-related fragments (pF262A, pF262B) and complete plasmids pF262C and pF262D were compared to complete *C. perfringens* plasmid sequences using the Artemis Comparison Tool, WebACT [20].

The pseudochromosome, plasmid fragments, and unplaced contigs were sent for automatic annotation through the Prokaryotic Genomes Automatic Annotation Pipeline (http://www.ncbi.nlm.nih.gov/genomes/static/Pipeline.html). The raw reads were submitted to the Sequence Read Archive database with Accession number SRP005501. The Whole Genome Shotgun project was deposited at DDBJ/EMBL/GenBank under the accession AFES00000000.

### Identification of unique nucleotide sequence using PanSeq

The annotated pseudochromosome, plasmid fragments and unassembled contigs were analyzed using PanSeq (http://lfz.corefacility.ca/panseq/), a software program that identifies unique nucleotide sequence by comparison to reference sequences [19]. Using the Novel Region Finder with a 1000 bp cutoff, *C. perfringens* F262 sequences were compared to the three available complete *C. perfringens* chromosome sequences (Strain 13, ATCC 13124 and SM101), the annotated and circularized *C. perfringens* plasmid sequences (pCPF4969, pCPF5603, pBCN5603, pSM101A, pSM101B, pCP13, pCW3, pIP404, pCP8533etx) and the fully annotated *C. perfringens* phage sequences (phage 39-O, phage phi3626 and phage phiSM101) in a variety of combinations. Strain 13, ATCC 13124 and SM101 are not the only *C. perfringens* genome sequences available, but at the time of analysis they were the only complete sequences available. Because we wanted to restrict the PanSeq analysis to plasmid or chromosomal DNA specifically, unassembled sequences, which do not distinguish chromosomal from plasmid data, were not included. The unique nucleotide sequences identified by PanSeq were matched to the corresponding ORFs and recorded.

### VirS frameshift mutation

To confirm the presence of a 2 bp deletion in *virS*, inward facing primers (inVirSF: 5′TTTTAAACATTTTCCCTCCAAGAAT3′ and inVirSR: 5′GGAAACAACAAAGAAACAGGTATAA3′) were designed and a touchdown PCR with an extension time of 2 min and final extension of 5 min was performed using a T-gradient Thermocycler (Biometra). The PCR products were purified using the QIAquick PCR Purification Kit (Qiagen) and sequenced at the University of Guelph Agriculture and Food Laboratory. To assess the expression of alpha-toxin and perfringolysin O, *C. perfringens* F262 was grown anaerobically overnight at 37°C on a sheep blood agar plate and examined for the presence of the double zone of hemolysis associated with these two toxins.

The ability of *C. perfringens* F262 to express beta2-toxin was also assessed. Immunohistochemistry of a paraffin cross-section from the F262 infected calf was performed using rabbit polyclonal antiserum prepared for us against recombinant consensus beta2-toxin (GenScript, Piscataway, NJ) and shown in unpublished studies to recognize atypical beta2-toxin. Serum from an unimmunized rabbit was used as a negative control. A tissue Gram stain was also performed.

## Supporting Information

Figure S1 **Contig alignments on** ***C. perfringens*** **F262 NcoI optical map.** *Footnote:* Optical mapping assembled approximately 3.1 Mbp of the estimated 3.46 Mbp chromosome. Contigs are designated as placed “*in silico*” based on comparison of optically mapped restriction sites to the contigs by use of the MapSolver™ software.
(TIF)

Table S1 **Virulence genes (Shimizu** ***et al.*** **, 2002) present in** ***Clostridium perfringens*** **F262.**
(DOC)

Table S2 **PanSeq results for Cfrag.**
(DOC)

Table S3 **PanSeq results for the** ***Clostridium perfringens*** **F262 pseudochromosome.**
(DOC)

Table S4 **PanSeq results for the orphan contigs.**
(DOC)
