# Supplementary figures and images for: Genome Sequencing and Analysis of a Type A Clostridium perfringens Isolate from a Case of Bovine Clostridial Abomasitis
Source: PLoS One. 2012 Mar 8;7(3):e32271. doi: 10.1371/journal.pone.0032271 (PMC3297601; doi:10.1371/journal.pone.0032271)

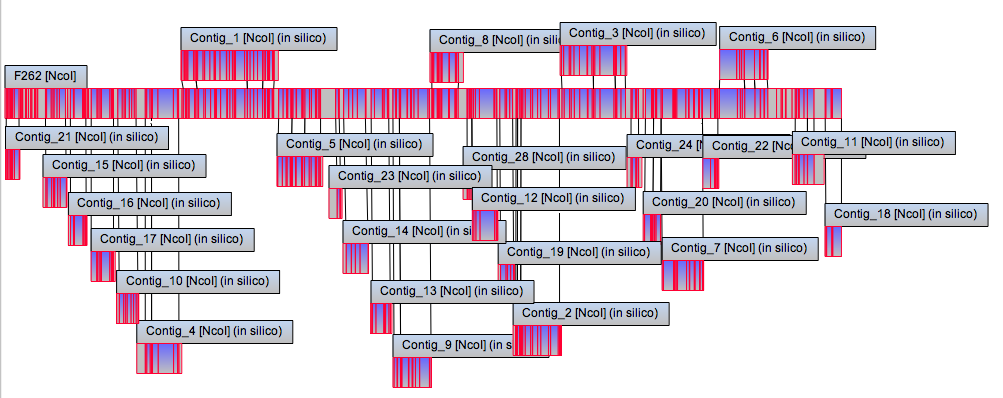

Supplement: Figure S1 — Contig alignments on C. perfringens F262 NcoI optical map. Footnote: Optical mapping assembled approximately 3.1 Mbp of the estimated 3.46 Mbp chromosome. Contigs are designated as placed “in silico” based on comparison of optically mapped restriction sites to the contigs by use of the MapSolver™ software. (TIF) [file pone.0032271.s001.tif]
