# Supplementary material for: Genome Sequencing and Analysis of a Type A Clostridium perfringens Isolate from a Case of Bovine Clostridial Abomasitis
Source: PLoS One. 2012 Mar 8;7(3):e32271. doi: 10.1371/journal.pone.0032271 (PMC3297601; doi:10.1371/journal.pone.0032271)
Supplement: Table S1 — Virulence genes (Shimizu et al. , 2002) present in Clostridium perfringens F262. (DOC) [file pone.0032271.s002.doc]

Table S1: Virulence genes (Shimizu *et al.,* 2002) present in *Clostridium perfringens* F262

| **Gene name** | **Product** | **Length (aa)** | **Presence in F262** |
| --- | --- | --- | --- |
| CPE0030 *hlyA* | Hemolysin-related protein | 421 | Contig_21 (HA1_00140) |
| CPE0036 *cpa* | Phospholipase C (alpha-toxin) | 398 | Contig_21 (HA1_00170) |
| CPE0163 *pfoA* | Perfringolysin O (theta-toxin) | 500 | Contig_15 (HA1_00843) |
| CPE0173 *colA* | Collagenase (kappa-toxin) | 1,104 | Contig_15 (HA1_00888) |
| CPE0191 *nagH* | Hyaluronidase (mu-toxin) | 1,628 | Contig_15 (HA1_00978) |
| CPE0378 | Myosin-crossreactive antigen | 597 | Contig_10 (HA1_01847) |
| CPE0437 *hlyB* | Hemolysin | 445 | Contig_4 (HA1_02192) |
| CPE0452 *entC* | Enterotoxin | 625 | Contig_4 (HA1_02267) |
| CPE0553 *nanJ* | Exo-alpha-sialidase | 1,173 | Contig_4 (HA1_02772) |
| CPE0606 *entD* | Enterotoxin | 635 | Contig_1 (HA1_03054) |
| CAA60796.1 *nanH* | Exo-alpha-sialidase | 694 | Contig_1 (HA1_03714) |
| CPE0737 | Fibronectin-binding protein | 220 | Contig_1 (HA1_03764) |
| CPE0846 | Alpha-clostripain | 524 | Contig_1 (HA1_04294) |
| CPE0881 *nagI* | Hyaluronidase (mu-toxin) | 1,297 | Contig_1 (353435 – 357329 bp, frameshift) |
| CPE1231 | Surface protein | 1,129 | Contig_9 (5′ end similar to HA1_07702) |
| CPE1234 *nagJ* | Hyaluronidase (mu-toxin) | 1,001 | Contig_8 (HA1_07722) |
| CPE1258 *entA* | Enterotoxin | 955 | Contig_8 (HA1_07852) |
| CPE1279 *nagK* | Hyaluronidase (mu-toxin) | 1,163 | Contig_8 (HA1_07917) |
| CPE1354 *entB* | Enterotoxin | 549 | Contig_28 (HA1_08317) |
| CPE1474 *hlyC* | Hemolysin | 215 | Contig_19 (HA1_09166) |
| CPE1523 *nagL* | Hyaluronidase (mu-toxin) | 1,127 | Contig_2 (HA1_09421) |
| CPE1818 *hlyD* | Hemolysin | 271 | Contig_3 (HA1_10896) |
| CPE1847 | Fibronectin-binding protein | 575 | Contig_3 (HA1_11041) |
| CPE1915 *hlyE* | Hemolysin III | 213 | Contig_3 (HA1_11371) |
