# Supplementary material for: Genome Sequencing and Analysis of a Type A Clostridium perfringens Isolate from a Case of Bovine Clostridial Abomasitis
Source: PLoS One. 2012 Mar 8;7(3):e32271. doi: 10.1371/journal.pone.0032271 (PMC3297601; doi:10.1371/journal.pone.0032271)
Supplement: Table S2 — PanSeq results for Cfrag. (DOC) [file pone.0032271.s003.doc]

Table S2: PanSeq results for Cfrag

The Cfrag sequence was compared against the *C. perfringens* Strain 13, ATCC 13124 and SM101 chromosomes using PanSeq and the unique regions identified. Regions that remained unique after comparison to all complete *C. perfringens* plasmid and phage sequences are bolded (every ORF but HA1_05497). Note: Cfrag was assembled by joining Contig_5, icontig00041, icontig00013 and Contig_23; the start of each contig in Cfrag have been marked with a grey shaded row.

| **Locus_tag** | **Size (aa)** | **Predicted Product (notes in brackets)** | **Hit Description** | **E-value** | **% Identity** | **Subcellular localization** | **Conserved domains** |
| --- | --- | --- | --- | --- | --- | --- | --- |
|  |  |  |  |  |  |  |  |
| **HA1_04797** | 315 | hypothetical protein | putative lipoprotein [*Clostridium perfringens* D str. JGS1721] | 2E-170 | 99% (312/315) | Cytoplasmic membrane (Y) 0.889 |  |
| **HA1_04807** | 264 | aminopeptidase (COG2234 Predicted aminopeptidases) | hypothetical protein AC3_1129 [*Clostridium perfringens* E str. JGS1987] | 2E-24 | 94% (58/64) | Cytoplasmic (N) | M20_dimer superfamily, Peptidase dimerization domain |
| **HA1_04812** | 85 | hypothetical protein | hypothetical protein CJD_1102 [*Clostridium perfringens* D str. JGS1721] | 6E-20 | 98% (51/69) | Cytoplasmic membrane (Y) 0.958 |  |
| **HA1_04817** | 124 | hypothetical protein | conserved hypothetical protein [*Clostridium perfringens* D str. JGS1721] | 2E-35 | 99% (80/81) | Cytoplasmic (N) |  |
| **HA1_04847** | 104 | hypothetical protein (COG1695 Predicted transcriptional regulators) | transcriptional regulator [*Clostridium perfringens* E str. JGS1987] | 8E-48 | 99% (103/104) | Cytoplasmic (N) | HTH DNA-binding domain, GntR family |
| **HA1_04852** | 219 | hypothetical protein | conserved hypothetical protein [*Clostridium perfringens* E str. JGS1987] | 2E-116 | 96% (211/219) | Cytoplasmic membrane (N) | COG4008 superfamily |
| **HA1_04857** | 145 | hypothetical protein | hypothetical protein Pat1_1499 [*Pseudoalteromonas atlantica* T6c] | 1E-12 | 33% (45/285) | Cytoplasmic (N) |  |
| **HA1_04897** | 921 | helicase, putative (COG3886 Predicted HKD family nuclease) | helicase [*Clostridium perfringens* D str. JGS1721] | 0 | 98% (901/963) | Cytoplasmic (N) | DEXDc superfamily, DEAD-like helicases superfamily |
| **HA1_04902** | 458 | hypothetical protein | hypothetical protein bcere0001_41620 [*Bacillus cereus* m1293] | 5E-18 | 24% (119/485) | Unknown (N) |  |
| **HA1_04907** | 114 | hypothetical protein | hypothetical protein bcere0001_41630 [*Bacillus cereus* m1293] | 3.5E-01 | 29% (24/90) | Cytoplasmic (N) |  |
| **HA1_04912** | 526 | peptidase C14, caspase catalytic subunit p20 (COG4249 uncharacterized protein containing caspase) | caspase domain [*Faecalibacterium prausnitzii* L2-6] | 1E-124 | 43% (221/511) | Unknown (N) | Caspase, interleukin-1 beta converting enzyme (ICE) homologues |
| **HA1_04917** | 942 | helicase (COG1112 Superfamily I DNA and RNA helicases and helicase subunits) | helicase [*Cellulophaga lytica* DSM 7489] | 0 | 60% (539/899) | Unknown (N) | UvrD-helicase superfamily, UvrD/REP helicase |
| **HA1_04922** | 117 | hypothetical protein (COG2856 Predicted Zn peptidase) | hypothetical protein CPF_1020 [*Clostridium perfringens* ATCC 13124] | 1E-26 | 51% (59/141) | Cytoplasmic (N) | DUF955 superfamily |
| **HA1_04927** | 179 | DNA-binding protein (COG1396 Predicted transcriptional regulators) | DNA-binding protein [*Clostridium perfringens* D str. JGS1721] | 1E-47 | 58% (103/180) | Cytoplasmic (N) | HTH XRE-family like proteins |
| **HA1_04932** | 642 | DNA polymerase (COG0749 DNA polymerase I - 3'-5' exonuclease and polymerase domains) | DNA-directed DNA polymerase [*Clostridium perfringens* B str. ATCC 3626] | 0 | 97% (625/642) | Cytoplasmic (N) | DNA_pol_A superfamily, DnaQ-like 3'-5' exonuclease domain superfamily |
| **HA1_04937** | 1020 | primase, putative (COG3378 Predicted ATPase) | phage/plasmid primase domain, P4 family [*Clostridium perfringens* B str. ATCC 3626] | 0 | 95% (968/995) | Cytoplasmic (N) | phage/plasmid primase, P4 family, C-terminal domain |
| **HA1_04942** | 197 | putative RNA polymerase sigma factor (COG1595 DNA-directed RNA polymerase specialized sigma subunit, sigma24 homolog) | RNA polymerase sigma factor, sigma-70 family [*Clostridium perfringens* B str. ATCC 3626] | 4E-105 | 98% (194/197) | Cytoplasmic (N) | Sigma70_r2 superfamily |
| **HA1_04947** | 125 | hypothetical protein | hypothetical protein AC1_1188 [*Clostridium perfringens* B str. ATCC 3626] | 9E-60 | 99% (124/125) | Cytoplasmic membrane (N) |  |
| **HA1_04952** | 61 | transposase | transposase [*Streptococcus suis* BM407] | 7E-15 | 71% (40/341) | Unknown (N) |  |
| **HA1_04982** | 510 | hypothetical protein | hypothetical protein CcarbDRAFT_0774 [*Clostridium carboxidivorans* P7] | 9E-31 | 29% (159/520) | Cytoplasmic (N) |  |
| **HA1_04987** | 206 | hypothetical protein | conserved hypothetical protein [*Roseburia intestinalis* L1-82] | 4.8E-02 | 28% (46/212) | Cytoplasmic (N) |  |
| **HA1_04992** | 805 | hypothetical protein (chromosome segregation ATPases) | putative RecF/RecN/SMC N domain protein [*Roseburia intestinalis* L1-82] | 3E-81 | 32% (271/805) | Cytoplasmic (N) | P-loop containing Nucleoside Triphosphate Hydrolases |
| **HA1_05072** | 230 | hypothetical protein (COG1583 Uncharacterized protein predicted to be involved in DNA repair (RAMP superfamily) | CRISPR-associated protein Cas6 [*Clostridium butyricum* E4 str. BoNT E BL5262] | 4E-66 | 50% (116/228) | Cytoplasmic (N) | CRISPR/Cas system-associated RAMP superfamily protein Cas6 |
| **HA1_05077** | 589 | hypothetical protein | CRISPR-associated protein, Csh1 family [*Clostridium butyricum* E4 strain BoNT E BL5262] | 3E-140 | 50% (297/586) | Cytoplasmic membrane (N) | CRISPR/Cas system-associated RAMP superfamily protein Cas8b |
| **HA1_05082** | 315 | hypothetical protein | conserved hypothetical protein [*Clostridium butyricum* E4 str. BoNT E BL5262] | 3E-121 | 67% (213/316) | Unknown (N) | CRISPR/Cas system-associated RAMP superfamily protein Cas7 |
| **HA1_05087** | 271 | hypothetical protein | CRISPR-associated protein Cas5 [*Clostridium butyricum* E4 str. BoNT E BL5262] | 3E-69 | 53% (145/262) | Unknown (N) | CRISPR/Cas system-associated RAMP superfamily protein Cas5 |
| **HA1_05092** | 910 | hypothetical protein (COG1203 Predicted helicases) | CRISPR-associated helicase Cas3 [*Clostridium butyricum* E4 str. BoNT E BL5262] | 0 | 49% (453/871) | Cytoplasmic (N) | DEXDc superfamily, DEAD-like helicases superfamily |
| **HA1_05097** | 163 | hypothetical protein (COG1468 RecB family exonuclease) | hypothetical protein CTC01464 [*Clostridium tetani* E88] | 2E-63 | 73% (120/163) | Unknown (N) | CRISPR/Cas system-associated RAMP superfamily protein Cas4 |
| **HA1_05102** | 332 | CRISPR-associated protein Cas1 (COG1518 Uncharacterized protein predicted to be involved in DNA repair) | CRISPR-associated helicase Cas1 [*Clostridium butyricum* E4 str. BoNT E BL5262] | 3E-156 | 79% (260/332) | Cytoplasmic membrane (N) | CRISPR/Cas system-associated protein Cas1 |
| **HA1_05107** | 96 | CRISPR-associated Cas2 family protein (COG1343 Uncharacterized protein predicted to be involved in DNA repair) | CRISPR-associated protein cas2 [*Anaerostipes* sp. 3_2_56FAA] | 6E-34 | 73% (70/96) | Cytoplasmic (N) | CRISPR/Cas system-associated protein Cas2 |
| **HA1_05472** | 139 | hypothetical protein | conserved hypothetical protein [*Clostridium perfringens* E str. JGS1987] | 1E-71 | 97% (135/139) | Unknown (N) |  |
| **HA1_05477** | 263 | hypothetical protein | hypothetical protein AC1_0642 [*Clostridium perfringens* B str. ATCC 3626] | 4E-139 | 98% (257/263) | Cytoplasmic membrane (N) |  |
| **HA1_05482** | 134 | PemK family protein (COG2337 Growth inhibitor) | PemK family protein [*Clostridium perfringens* B str. ATCC 3626] | 1E-70 | 99% (133/134) | Unknown (N) | PemK-like superfamily, PemK-like protein |
| **HA1_05487** | 89 | hypothetical protein | conserved domain protein [*Clostridium perfringens* D str. JGS171] | 5E-43 | 97% (86/97) | Cytoplasmic (N) |  |
| **HA1_05492** | 375 | hypothetical protein (COG2340 Uncharacterized protein with SCP/PR1 domains) | putative conserved hypothetical protein [*Clostridium perfringens* E str. JGS1987] | 0 | 91% (344/377) | Unknown (Y) 0.921 | SCP superfamily, SCP-like extracellular protein domain, found in eukaryotes and prokaryotes |
| HA1_05497 | 116 | hypothetical protein | hypothetical protein AC3_A0275 [*Clostridium perfringens* E str. JGS1987] | 1E-58 | 97% (112/116) | Unknown (N) | YopX superfamily |
| **HA1_05522** | 35 | hypothetical protein | hypothetical protein AC3_A0277 [*Clostridium perfringens* E str. JGS1987] | 3E-05 | 82% (27/43) | Cytoplasmic membrane (Y) 0.168 |  |
| **HA1_05527** | 82 | hypothetical protein | conserved hypothetical protein [*Clostridium perfringens* B str. ATCC 3626] | 4E-36 | 99% (81/82) | Cytoplasmic (N) |  |
| **HA1_05532** | 198 | hypothetical protein (COG3764 Sortase, surface protein transpeptidase) | sortase family protein [*Clostridium perfringens* B str. ATCC 3626] | 1E-105 | 99% (197/198) | Unknown (Y) 0.983 | Sortase superfamily |
| **HA1_05537** | 102 | hypothetical protein | hypothetical protein AC1_0652 [*Clostridium perfringens* B str. ATCC 3626] | 7E-48 | 100% (102/102) | Unknown (Y) 0.150 |  |
| **HA1_05542** | 31 | hypothetical protein | hypothetical protein AC1_A0281 [*Clostridium perfringens* B str. ATCC 3626] | 1E-03 | 73% (22/52) | Unknown (N) |  |
| **HA1_05547** | 126 | single-strand DNA-binding protein (COG0629 Single-stranded DNA-binding protein) | single-strand binding protein family [*Clostridium perfringens* E str. JGS1987] | 1E-62 | 96% (121/126) | Cytoplasmic (N) | Replication protein A, class 2b aminoacyl-tRNA synthetases |
| **HA1_05552** | 71 | hypothetical protein (COG1476 Predicted transcriptional regulators) | transcriptional regulator, XRE family [*Clostridium perfringens* E str. JGS1987] | 5E-29 | 97% (69/71) | Cytoplasmic (N) | HTH_XRE superfamily |
| **HA1_05557** | 78 | hypothetical protein (COG1396 Predicted transcriptional regulators | cI2009 [*Clostridium perfringens* E str. JGS1987] | 3E-33 | 95% (74/78) | Unknown (N) | HTH XRE-family like proteins |
| **HA1_05562** | 69 | hypothetical protein | conserved hypothetical protein [*Clostridium perfringens* E str. JGS1987] | 2E-29 | 99% (68/69) | Unknown (N) |  |
| **HA1_05567** | 61 | hypothetical protein | hypothetical protein CJD_A0260 [*Clostridium perfringens* D str. JGS1721] | 1E-10 | 73% (46/63) | Cytoplasmic (N) |  |
| **HA1_05572** | 86 | hypothetical protein | conserved hypothetical protein [*Clostridium perfringens* D str. JGS1721] | 7E-19 | 70% (60/85) | Cytoplasmic membrane (N) |  |
| **HA1_05577** | 731 | type IV secretory pathway, VirD4 component (COG3505 Type IV secretory pathway, VirD4 components) | TraG/TraD family [*Clostridium perfringens* D str. JGS1721] | 0 | 90% (661/731) | Cytoplasmic membrane (Y) 0.988 | P-loop containing Nucleoside Triphosphate Hydrolases |
| **HA1_05582** | 626 | hypothetical protein | conserved hypothetical protein [*Clostridium perfringens* D str. JGS1721] | 0 | 68% (430/623) | Unknown (N) |  |
| **HA1_05587** | 94 | hypothetical protein | conserved hypothetical protein [*Clostridium perfringens* D str. JGS1721] | 4E-36 | 80% (75/95) | Cytoplasmic membrane (N) |  |
| **HA1_05592** | 220 | hypothetical protein | conserved hypothetical protein [*Clostridium perfringens* D str. JGS1721] | 1E-76 | 86% (189/220) | Unknown (N) |  |
| **HA1_05597** | 629 | hypothetical protein (COG3451 Type IV secretory pathway, VirB4 components) | conserved hypothetical protein [*Clostridium perfringens* D str. JGS1721] | 0 | 92% (580/629) | Cytoplasmic (N) | P-loop containing Nucleoside Triphosphate Hydrolases |
| **HA1_05602** | 199 | hypothetical protein | hypothetical protein CJD_A0253 [*Clostridium perfringens* D str. JGS1721] | 5E-80 | 81% (162/199) | Unknown (Y) 0.398 |  |
| **HA1_05607** | 383 | cell wall-binding protein (Soluble lytic murein transglycosylase and related regulatory proteins, some contain LysM/invasin domains) | probable cell wall-binding protein [*Clostridium perfringens* D str. JGS1721] | 0 | 86% (323/374) | Cell wall (Y) 0.994 | NLPC_P60 superfamily |
| **HA1_05612** | 281 | hypothetical protein | conserved hypothetical protein [*Clostridium perfringens* D str. JGS1721] | 4E-118 | 80% (224/283) | Unknown (Y) 0.011 |  |
| **HA1_05617** | 95 | hypothetical protein (COG0542 ATPases with chaperone activity, ATP-binding subunit) | conserved hypothetical protein [*Clostridium perfringens* D str. JGS1721] | 4E-39 | 95% (87/97) | Cytoplasmic (N) |  |
| **HA1_05622** | 134 | hypothetical protein | conserved hypothetical protein [*Clostridium perfringens* D str. JGS1721] | 1E-62 | 93% (124/134) | Cytoplasmic (N) |  |
| **HA1_05627** | 375 | hypothetical protein | conserved hypothetical protein [*Clostridium perfringens* D str. JGS1721] | 0 | 92% (344/375) | Unknown (N) |  |
| **HA1_05632** | 68 | hypothetical protein | conserved hypothetical protein [*Clostridium perfringens* E str. JGS1987] | 2E-26 | 94% (64/68) | Cytoplasmic (N) |  |
| **HA1_05637** | 608 | hypothetical protein (COG1357 Uncharacterized low-complexity proteins) | conserved hypothetical protein [*Clostridium perfringens* D str. JGS1721] | 0 | 86% (420/819) | Cytoplasmic (N) | Pentapeptide superfamily, DUF955 |
| **HA1_05642** | 102 | hypothetical protein | conserved hypothetical protein [*Clostridium perfringens* E str. JGS1987] | 8E-50 | 100% (102/102) | Unknown (N) |  |
| **HA1_05647** | 98 | hypothetical protein | conserved hypothetical protein [*Clostridium perfringens* D str. JGS1721] | 6E-47 | 95% (93/98) | Unknown (N) |  |
| **HA1_05652** | 67 | hypothetical protein | conserved hypothetical protein [*Clostridium perfringens* E str. JGS1987] | 4E-27 | 93% (62/67) | Extracellular (N) |  |
| **HA1_05657** | 49 | hypothetical protein | hypothetical protein AC1_A0683 [*Clostridium perfringens* B str. ATCC 3626] | 2E-14 | 98% (48/49) | Cytoplasmic (N) |  |
| **HA1_05662** | 251 | hypothetical protein | hypothetical protein Cloce1_0790 [*Clostridium cellulovorans* 743B] | 1E-98 | 71% (179/250) | Cytoplasmic (N) |  |
| **HA1_05667** | 397 | partition protein (COG1475 Predicted transcriptional regulators) | partition protein [*Clostridium perfringens* B str. ATCC 3626] | 0 | 97% (384/397) | Cytoplasmic (N) | ParBc superfamily, ParB-like nuclease domain |
| **HA1_05672** | 250 | Soj protein (COG1192 ATPases involved in chromosome partitioning) | sporulation initiation inhibitor protein soj [*Clostridium perfringens* D str. JGS1721) | 1E-137 | 98% (246/251) | Cytoplasmic membrane (N) | Ras_like_GTPase superfamily |
| **HA1_05677** | 74 | hypothetical protein | conserved hypothetical protein [*Clostridium perfringens* E str. JGS1987] | 4E-30 | 92% (68/74) | Unknown (N) |  |
| **HA1_05682** | 450 | transposase IS116/IS110/IS902 family protein (COG3547 Transposase and inactivated derivatives) | putative transposase [*Clostridium perfringens* E str. JGS1987] | 0 | 97% (435/450) | Cytoplasmic (N) | Transposase_20 superfamily |
