# Supplementary material for: Genome Sequencing and Analysis of a Type A Clostridium perfringens Isolate from a Case of Bovine Clostridial Abomasitis
Source: PLoS One. 2012 Mar 8;7(3):e32271. doi: 10.1371/journal.pone.0032271 (PMC3297601; doi:10.1371/journal.pone.0032271)
Supplement: Table S3 — PanSeq results for the Clostridium perfringens F262 pseudochromosome. (DOC) [file pone.0032271.s004.doc]

Table S3: PanSeq results for the *Clostridium perfringens* F262 pseudochromosome

The *C. perfringens* F262 pseudochromosome was compared against the *C. perfringens* Strain 13, ATCC 13124 and SM101 chromosomes using PanSeq and the unique regions identified. Regions that remained unique after comparison to all complete *C. perfringens* plasmid and phage sequences are bolded. Everything remained unique except for some ORFs within Contig_14 and contig00062.

| **Contig** | **Locus_tag** | **Size (aa)** | **Predicted Product (notes in brackets)** | **Hit Description** | **E-value** | **% identity** | **Subcellular localization** | **Conserved domains** |
| --- | --- | --- | --- | --- | --- | --- | --- | --- |
|  |  |  |  |  |  |  |  |  |
| Contig_1 | **HA1_03124** | 353 | hypothetical protein | conserved hypothetical protein [*Clostridium perfringens* CPE str. F4969] | 0 | 96% (341/353) | Cytoplasmic (N) | RgpF superfamily |
|  | **HA1_03094** | 361 | sugar transferase family protein (COG2148 sugar transferases involved in LPS synthesis) | glycosyltransferase [*Clostridium perfringens* CPE str. F4969] | 0 | 336/364) | Unknown (N) | GT1_YqgM_like, Glycosyltransferase_GTB_type superfamily, RfaG |
|  | **HA1_03134** | 417 | hypothetical protein | hypothetical protein AC7_0635 [*Clostridium perfringens* NCTC 8239] | E-150 | 71% (298/417) | Cytoplasmic membrane (Y) 0.269 | |
|  | **HA1_03139** | 274 | putative lipopolysaccharide biosynthesis protein (COG3475) | LicD-related protein [*Clostridium perfringens* D str. JGS1721] | E-158 | 100% (274/274) | Unknown (N) | LicD superfamily |
|  | **HA1_03154** | 511 | hypothetical protein (COG5263 glucan-binding domain, YG repeat) | hypothetical protein CPE0625 [*Clostridium perfringens* Strain 13] | 0 | 67% (338/550) | Extracellular (Y) 1.000 | Peptidase_M14_like superfamily, COG5263, glucan_65_rpt |
|  | **HA1_03159** | 811 | hypothetical protein (COG5263 glucan-binding domain, YG repeat) | cell wall binding repeat protein [*Clostridium perfringens* CPE str. F4969] | 0 | 90% (771/898) | Cytoplasmic membrane (Y) 1.000 | COG3786 superfamily, glucan_65_rpt |
|  | **HA1_03324** | 659 | glycosyl transferase, group 1 family (COG0438 glycosyltransferase) | glycosyl transferase, group 1 family protein [*Clostridium perfringens* E str. JGS1987] | 1E-49 | 32% (124/382) | Unknown (N) | GT1_WabH_like, Glycosyltransferase_GTB_type superfamily, RfaG |
|  | **HA1_04104** | 286 | transcriptional regulator, LacI family (COG1609) | transcriptional regulator [*Enterococcus casseliflavus* ATCC 12755] | 1E-51 | 37% (109/341) | Cytoplasmic (N) | PBP1_LacI_sugar_binding_like, Periplasmic_Binding_Protein_Type_1 superfamily, HTH_LacI, HTH_LacI superfamily |
|  | **HA1_04304** | 305 | hypothetical protein | hypothetical protein DICPUDRAFT_146816 [*Dictyostelium purpureum*] | 0.006 | 25% (52/430) | Unknown (Y) 0.385 |  |
|  | **HA1_04309** | 76 | hypothetical protein | No hits found | |  | Unknown (N) |  |
|  | **HA1_04314** | 248 | hypothetical protein | hypothetical protein [*Staphylococcus cohnii*] | 4E-07 | 28% (53/372) | Cytoplasmic (N) |  |
|  |  |  |  |  |  |  |  |  |
| Contig_10 | **HA1_01822** | 241 | hypothetical protein | hypothetical protein TcarDRAFT_1572 [*Thermosinus carboxydivorans* Nor1] | 4E-37 | 33% (80/242) | Cytoplasmic membrane (N) |  |
|  | **HA1_01877** | 211 | putative RNA polymerase sigma factor SigI (COG1191 DNA-directed RNA polymerase specialized sigma subunit) | putative DNA-directed RNA polymerase sigma factor [*Clostridium perfringens* D str. JGS1721] | 4E-94 | 86% (182/219) | Cytoplasmic (N) | PRK08311 |
|  | **HA1_01882** | 348 | hypothetical protein | hypothetical protein CJD_0460 [*Clostridium perfringens* D str. JGS1721] | E-143 | 86% (265/348) | Cell wall (N) |  |
|  | **HA1_01887** | 211 | hypothetical protein | conserved hypothetical protein [*Clostridium perfringens* D str. JGS1721] | E-117 | 100% (211/232) | Unknown (N) | PolyPPase_VTC_like, CYTH-like_Pase superfamily |
|  | **HA1_01892** | 211 | hypothetical protein | conserved hypothetical protein [*Clostridium perfringens* D str. JGS1721] | 7E-84 | 80% (169/226) | Cytoplasmic membrane (N) |  |
|  | **HA1_01897** | 183 | resolvase domain (COG1961 site-specific recombinase DNA, invertase) | Resolvase domain [*Ethanoligenens harbinense* YUAN-3] | 1E-39 | 49% (89/188) | Unknown (N) | SR_ResInv, Ser_Recombinase superfamily, HTH_Hin_like superfamily, mpi |
|  | **HA1_01902** | 230 | hypothetical protein (COG5036 SPX domain-containing protein involved in vacuolar polyphosphate accumulation) | conserved hypothetical protein [*Clostridium perfringens* D str. JGS1721] | E-129 | 99% (229/230) | Unknown (N) | PolyPPase_VTC_like, CYTH-like_Pase superfamily |
|  | **HA1_01907** | 211 | hypothetical protein | tubulin/FtsZ, GTPase [*Clostridium perfringens* D str. JGS1721] | E-100 | 92% (195/222) | Cytoplasmic membrane (N) |  |
|  | **HA1_01912** | 436 | hypothetical protein | CotH protein [*Clostridium perfringens* D str. JGS1721] | 0 | 95% (418/497) | Unknown (Y) 0.235 | CotH |
|  | **HA1_01917** | 685 | hypothetical protein (COG1702 phosphate starvation-inducible protein PhoH, predicted ATPase) | conserved hypothetical protein [*Clostridium perfringens* D str. JGS1721] | 0 | 96% (663/685) | Unknown (N) |  |
|  | **HA1_01922** | 361 | HEAT repeat-containing PBS lyase (COG1413 HEAT repeat) | heat repeat domain protein [*Clostridium perfringens* D str. JGS1721] | E-172 | 93% (314/377) | Unknown (N) |  |
|  | **HA1_01927** | 466 | glycosyl transferase family protein (COG1215 glycosyltransferases, probably involved in cell wall biogenesis) | chitin synthase [*Clostridium perfringens* D str. JGS1721] | 0 | 90% (424/466) | Cytoplasmic membrane (N) | CESA_like cellulose superfamily |
|  |  |  |  |  |  |  |  |  |
| Contig_12 | **HA1_08482** | 254 | phage-related terminase small subunit-like protein (COG5484 uncharacterized conserved protein) | phage-related terminase small subunit-like protein [*Clostridium beijerinckii* NCIMB 8052] | 1E-64 | 53% (130/260) | Cytoplasmic (N) | Phage_terminase superfamily |
|  | **HA1_08487** | 215 | hypothetical protein | hypothetical protein CKL_1872 [*Clostridium kluyveri* DSM 555] | 8E-06 | 25% (52/660) | Cytoplasmic (N) |  |
|  | **HA1_08492** | 436 | hypothetical protein | hypothetical protein CKL_1872 [*Clostridium kluyveri* DSM 555] | E-125 | 54% (237/660) | Cytoplasmic (N) |  |
|  | **HA1_08502** | 198 | hypothetical protein | No hits found | |  | Unknown (N) |  |
|  | **HA1_08507** | 231 | hypothetical protein | No hits found | |  | Unknown (N) |  |
|  | **HA1_08527** | 62 | hypothetical protein | hypothetical protein AC1_1083 [*Clostridium perfringens* B str. ATCC 3626] | 5E-27 | 100% (62/62) | Cytoplasmic (N) |  |
|  | **HA1_08532** | 286 | hypothetical protein | hypothetical protein AC1_1082 [*Clostridium perfringens* B str. ATCC 3626] | E-131 | 87% (249/303) | Cytoplasmic (N) | DUF1351 |
|  | **HA1_08537** | 211 | putative phage essential recombination function | peptidyl-prolyl cis-trans isomerase family protein [*Clostridium perfringens* C str. JGS1495] | E-103 | 89% (189/217) | Cytoplasmic (N) | ERF superfamily |
|  | **HA1_08542** | 61 | hypothetical protein | conserved hypothetical protein [*Clostridium perfringens* B str. ATCC 3626] | 6E-31 | 98% (60/62) | Cytoplasmic (N) |  |
|  | **HA1_08547** | 43 | hypothetical protein | conserved hypothetical protein [*Clostridium perfringens* B str. ATCC 3626] | 1E-09 | 97% (34/79) | Unknown (N) |  |
|  | **HA1_08552** | 36 | hypothetical protein | No hits found | |  | Cytoplasmic membrane (Y) 0.062 | |
|  | **HA1_08557** | 54 | hypothetical protein | hypothetical protein AC1_1078 [*Clostridium perfringens* B str. ATCC 3626] | 5E-14 | 97% (37/54) | Extracellular (N) |  |
|  | **HA1_08562** | 61 | hypothetical protein | conserved domain protein [*Clostridium perfringens* B str. ATCC 3626] | 2E-27 | 98% (60/69) | Unknown (N) |  |
|  | **HA1_08567** | 94 | hypothetical protein | hypothetical protein AC1_1077 [*Clostridium perfringens* B str. ATCC 3626] | 2E-44 | 95% (90/94) | Cytoplasmic (N) |  |
|  | **HA1_08572** | 59 | hypothetical protein | conserved domain protein [*Clostridium perfringens* B str. ATCC 3626] | 2E-22 | 93% (55/59) | Cytoplasmic membrane (Y) 0.02 | |
|  | **HA1_08577** | 77 | hypothetical protein | conserved hypothetical protein [*Clostridium perfringens* B str. ATCC 3626] | 6E-37 | 100% (77/77) | Unknown (N) |  |
|  | **HA1_08587** | 61 | hypothetical protein | conserved hypothetical protein [*Clostridium botulinum* E1 str. BoNT E Beluga] | 3E-03 | 46% (19/67) | Cytoplasmic (N) |  |
|  | **HA1_08592** | 170 | transcriptional regulator, Cro/CI family protein (COG1396 predicted transcriptional regulators) | transcriptional regulator, Cro/CI family [*Clostridium botulinum* E3 str. Alaska E43] | 5E-30 | 51% (81/160) | Cytoplasmic (N) | HTH_XRE-family like proteins |
|  | **HA1_08597** | 154 | hypothetical protein | hypothetical protein CPF_0927 [*Clostridium perfringens* ATCC 13124] | 1E-44 | 55% (85/154) | Cytoplasmic (N) | DUF955 superfamily |
|  |  |  |  |  |  |  |  |  |
| Contig_14 | **HA1_06057** | 180 | RNA polymerase factor sigma-70 (COG1595 DNA-directed RNA polymerase specialized sigma subunit, sigma24 homolog) | RNA polymerase sigma-70 factor [*Clostridium perfringens* D str. JGS1721] | 1E-98 | 100% (180/180) | Cytoplasmic (N) | Sigma70_r2 superfamily, Sigma70_r4 superfamily, PRK06811 |
|  | **HA1_06062** | 436 | hypothetical protein | hypothetical protein CJD_1308 [*Clostridium perfringens* D str. JGS1721] | 0 | 86% (377/443) | Cytoplasmic (N) |  |
|  | **HA1_06197** | 490 | resolvase family protein (COG1961 site-specific recombinases, DNA invertase) | site-specific recombinase [*Clostridium perfringens* D str. JGS1721] | 0 | 88% (434/490) | Unknown (N) | Ser_Recombinase, Ser_Recombinase superfamily, PinR |
|  | **HA1_06202** | 123 | helix-turn-helix domain-containing protein (COG1396 predicted transcriptional regulators) | phage transcriptional regulator [*Clostridium perfringens* D str. JGS1721] | 3E-61 | 100% (123/123) | Unknown (N) | HTH_XRE superfamily |
|  | **HA1_06207** | 81 | helix-turn-helix domain-containing protein (COG1396 predicted transcriptional regulators) | helix-turn-helix motif [*Clostridium perfringens* D str. JGS1721] | 1E-37 | 98% (80/81) | Unknown (N) | HTH_XRE superfamily |
|  | **HA1_06212** | 37 | hypothetical protein | No hits found | |  | Extracellular (N) |  |
|  | **HA1_06217** | 65 | hypothetical protein | hypothetical protein CJD_1341 [*Clostridium perfringens* D str. JGS1721] | 1E-16 | 72% (44/64) | Unknown (N) |  |
|  | **HA1_06222** | 44 | hypothetical protein | hypothetical protein AC1_2251 [*Clostridium perfringens* B str. ATCC 3626] | 3E-11 | 80% (34/46) | Cytoplasmic (N) |  |
|  | **HA1_06227** | 59 | hypothetical protein | conserved domain protein [*Clostridium perfringens* B str. ATCC 3626] | 1E-12 | 97% (37/59) | Cytoplasmic membrane (N) |  |
|  | **HA1_06232** | 60 | hypothetical protein | hypothetical protein AC1_2248 [*Clostridium perfringens* B str. ATCC 3626] | 5E-14 | 68% (39/62) | Cytoplasmic (N) |  |
|  | **HA1_06237** | 156 | hypothetical protein | No hits found | |  | Cytoplasmic (N) |  |
|  | **HA1_06242** | 221 | hypothetical protein | No hits found | |  | Unknown (Y) 0.748 |  |
|  | **HA1_06247** | 50 | hypothetical protein | hypothetical protein AC1_2247 [*Clostridium perfringens* B str. ATCC 3626] | 6E-07 | 81% (27/51) | Extracellular (N) |  |
|  | **HA1_06252** | 100 | hypothetical protein (COG0099 ribosomal protein S13) | hypothetical protein AC1_2246 [*Clostridium perfringens* B str. ATCC 3626] | 2E-07 | 35% (32/102) | Cytoplasmic (N) |  |
|  | **HA1_06257** | 109 | hypothetical protein | Gp31 protein [*Clostridium perfringens* B str. ATCC 3626] | 8E-26 | 69% (58/108) | Cytoplasmic (N) |  |
|  | **HA1_06262** | 62 | hypothetical protein | conserved hypothetical protein [*Clostridium perfringens* B str. ATCC 3626] | 2E-26 | 98% (61/62) | Cytoplasmic (N) |  |
|  | **HA1_06267** | 42 | hypothetical protein | hypothetical protein bthur0004_55680 [*Bacillus thuringiensis* serovar sotto str. T04001] | 3E-04 | 47% (21/259) | Unknown (N) |  |
|  | **HA1_06272** | 226 | hypothetical protein | hypothetical protein CPR_C0032 [*Clostridium* phage phiSM101] | 6E-24 | 61% (60/114) | Unknown (N) |  |
|  | **HA1_06277** | 262 | hypothetical protein | conserved hypothetical protein [*Clostridium perfringens* B str. ATCC 3626] | E-124 | 88% (221/262) | Cytoplasmic (N) |  |
|  | **HA1_06282** | 90 | hypothetical protein (COG0640 predicted transcriptional regulators) | transcription regulator [*Clostridium perfringens* B str. ATCC 3626] | 5E-43 | 98% (89/90) | Cytoplasmic (N) |  |
|  | **HA1_06287** | 60 | hypothetical protein | hypothetical protein AC1_2241 [*Clostridium perfringens* B str. ATCC 3626] | 6E-26 | 96% (58/60) | Unknown (N) |  |
|  | **HA1_06292** | 82 | glutamate racemase | hypothetical protein AC1_2240 [*Clostridium perfringens* B str. ATCC 3626] | 7E-35 | 93% (77/82) | Cytoplasmic (N) |  |
|  | **HA1_06297** | 80 | hypothetical protein | hypothetical protein AC1_2239 [*Clostridium perfringens* B str. ATCC 3626] | 8E-36 | 88% (71/80) | Cytoplasmic (N) |  |
|  | **HA1_06302** | 51 | hypothetical protein | conserved hypothetical protein [*Clostridium perfringens* B str. ATCC 3626] | 9E-22 | 96% (49/51) | Unknown (N) |  |
|  | **HA1_06307** | 101 | hypothetical protein | hypothetical protein CPR_C0027 [*Clostridium* phage phiSM101] | 3E-20 | 54% (52/338) | Unknown (N) |  |
|  | **HA1_06312** | 95 | hypothetical protein | hypothetical protein CPR_C0027 [*Clostridium* phage phiSM101] | 5E-04 | 57% (23/338) | Cytoplasmic (N) |  |
|  | **HA1_06317** | 64 | hypothetical protein | conserved domain protein [*Clostridium perfringens* D str. JGS1721] | 1E-16 | 65% (42/75) | Cytoplasmic (N) |  |
|  | **HA1_06322** | 144 | hypothetical protein (phosphoribosylaminoimidazolesuccinocarboxamide, SAICAR) | hypothetical protein CJD_1357 [*Clostridium perfringens* D str. JGS1721] | 1E-17 | 44% (51/108) | Cytoplasmic (N) | YopX superfamily |
|  | **HA1_06327** | 90 | hypothetical protein | hypothetical protein CPR_C0025 [*Clostridium* phage phiSM101] | 2E-32 | 73% (66/90) | Unknown (N) |  |
|  | **HA1_06332** | 152 | phage protein | phage protein [*Clostridium botulinum* A str. ATCC 3502] | 2E-18 | 38% (63/141) | Cytoplasmic (N) |  |
|  | **HA1_06337** | 149 | hypothetical protein | probable sigma factor [*Clostridium* phage phiSM101] | 8E-139 | 55% (82/153) | Cytoplasmic (N) |  |
|  | **HA1_06342** | 46 | hypothetical protein | conserved hypothetical protein [*Clostridium perfringens* B str. ATCC 3626] | 1E-14 | 95% (39/45) | Unknown (N) |  |
|  | **HA1_06347** | 117 | hypothetical protein | hypothetical protein AC1_2228 [*Clostridium perfringens* B str. ATCC 3626] | 5E-47 | 82% (97/117) | Cytoplasmic (N) |  |
|  | **HA1_06352** | 98 | hypothetical protein | conserved hypothetical protein [*Clostridium perfringens* B str. ATCC 3626] | 1E-38 | 78% (77/98) | Cytoplasmic (N) |  |
|  | **HA1_06357** | 62 | hypothetical protein | hypothetical protein AC1_2226 [*Clostridium perfringens* B str. ATCC 3626] | 1E-15 | 67% (42/59) | Cytoplasmic (N) |  |
|  | **HA1_06362** | 88 | hypothetical protein | hypothetical protein Cphy_2966 [*Clostridium phytofermentans* ISDq] | 8E-36 | 81% (70/86) | Unknown (N) |  |
|  | **HA1_06367** | 127 | putative electron transporter (COG1403 restriction endonuclease) | phage endonuclease [*Clostridium perfringens* B str. ATCC 3626] | 7E-62 | 86% (110/127) | Cytoplasmic (N) | HNHc superfamily |
|  | **HA1_06372** | 64 | hypothetical protein | hypothetical protein AC1_2223 [*Clostridium perfringens* B str. ATCC 3626] | 1E-18 | 73% (47/64) | Cytoplasmic (N) |  |
|  | **HA1_06377** | 178 | phage terminase, small subunit (P27 family protein) | putative phage terminase, small subunit [*Clostridium perfringens* B str. ATCC 3626] | 2E-84 | 92% (164/178) | Cytoplasmic (N) | Terminase_4 superfamily |
|  | **HA1_06382** | 566 | putative terminase, large subunit (COG4626 phage terminase-like protein, large subunit) | putative phage terminase, large subunit [*Clostridium perfringens* B str. ATCC 3626] | 0 | 97% (552/566) | Cytoplasmic (N) | Terminase_1 superfamily |
|  | **HA1_06387** | 384 | HK97 family phage portal protein (COG4695 phage-related protein) | phage portal protein, HK97 family [*Clostridium perfringens* B str. ATCC 3626] | 0 | 88% (338/384) | Unknown (N) | Phage_portal superfamily |
|  | **HA1_06392** | 255 | putative Clp protease (COG0740 protease subunit of ATP-dependent Clp proteases) | serine protease [*Clostridium perfringens* B str. ATCC 3626] | E-124 | 92% (222/255) | Unknown (N) | ClpP, crotonase-like superfamily |
|  | **HA1_06397** | 378 | putative phage capsid protein | phage prohead protease [*Clostridium perfringens* B str. ATCC 3626] | 0 | 93% (352/378) | Unknown (N) | Phage_capsid superfamily |
|  | **HA1_06402** | 62 | hypothetical protein | phage protein [*Clostridium perfringens* B str. ATCC 3626] | 6E-21 | 98% (52/54) | Unknown (N) |  |
|  | **HA1_06407** | 97 | hypothetical protein (COG1055 Na+/H+ antiporter NhaD and related arsenite) | conserved hypothetical protein [*Clostridium perfringens* B str. ATCC 3626] | 7E-46 | 97% (95/97) | Unknown (N) | gp6, gp6_gp15_like superfamily |
|  | **HA1_06412** | 114 | hypothetical protein | putative phage head-tail adaptor [*Clostridium perfringens* B str. ATCC 3626] | 3E-47 | 85% (98/114) | Cytoplasmic (N) | Phage_H_T_join superfamily |
|  | **HA1_06417** | 139 | hypothetical protein | hypothetical protein AC1_2214 [*Clostridium perfringens* B str. ATCC 3626] | 7E-65 | 87% (122/139) | Unknown (N) |  |
|  | **HA1_06422** | 103 | hypothetical protein | hypothetical protein AC1_2213 [*Clostridium perfringens* B str. ATCC 3626] | 1E-51 | 98% (101/103) | Unknown (N) |  |
|  | **HA1_06427** | 193 | hypothetical protein | phage major tail protein, Phi13 family [*Clostridium perfringens* B str. ATCC 3626] | 2E-89 | 85% (165/193) | Unknown (N) | maj_tail_phi13 superfamily |
|  | **HA1_06432** | 107 | hypothetical protein | hypothetical protein AC1_2211 [*Clostridium perfringens* B str. ATCC 3626] | 4E-51 | 98% (105/107) | Cytoplasmic (N) |  |
|  | **HA1_06437** | 1391 | TP901 family phage tail tape measure protein (phage-related tail protein) | putative phage tail tape measure protein, family, core region domain protein [*Clostridium perfringens* B str. ATCC 3626] | 0 | 92% (1282/1391) | Cell wall (N) | chromosome segregation protein SMC, tape_meas_TP901, SMC, DNA topoisomerase subtype IIA |
|  | HA1_06442 | 216 | tail component family protein | conserved hypothetical protein [*Clostridium perfringens* B str. ATCC 3626] | E-108 | 91% (198/217) | Cytoplasmic (N) | Sipho_tail superfamily |
|  | **HA1_06447** | 97 | hypothetical protein | hypothetical protein CPR_C0002 [*Clostridium* phage phiSM101] | 1E-33 | 78% (76/97) | Unknown (N) |  |
|  | **HA1_06452** | 78 | hypothetical protein | hypothetical protein AC1_2205 [*Clostridium perfringens* B str. ATCC 3626] | 2E-24 | 74% (58/78) | Extracellular (N) |  |
|  | HA1_06457 | 1832 | KID repeat family protein (COG1357 uncharacterized low-complexity proteins) | KID repeat family protein [*Clostridium perfringens* CPE str. F4969] | 0 | 82% (972/2408) | Cell wall (N) | MPP_superfamily, DUF1142, adenine deaminase, Ttg2C, AtpF |
|  |  |  |  |  |  |  |  |  |
| Contig_15 | **HA1_00708** | 136 | hypothetical protein (COG1403 restriction endonuclease) | gp75 [*Mycobacterium* phage Tweety] | 0.035 | 42% (17/186) | Unknown (N) |  |
|  | **HA1_00713** | 361 | hypothetical protein | hypothetical protein Npun_F4488 [*Nostoc punctiforme* PCC 73102] | 3E-11 | 22% (71/343) | Cytoplasmic (N) | DGQHR superfamily |
|  | **HA1_00718** | 136 | hypothetical protein | No hits found | |  | Cytoplasmic membrane (Y) 0.998 | |
|  | **HA1_00723** | 61 | recombinase (COG1961 site-specific recombinases, DNA invertase) | Recombinase [*Sebaldella termitidis* ATCC 33386] | 4E-07 | 48% (27/496) | Unknown (N) | Recombinase superfamily |
|  | **HA1_00728** | 61 | hypothetical protein | No hits found | |  | Unknown (N) |  |
|  |  |  |  |  |  |  |  |  |
| Contig_28 | **HA1_08342** | 341 | hypothetical protein | hypothetical protein AC3_0058 [*Clostridium perfringens* E str. JGS1987] | 5E-33 | 31% (94/304) | Cytoplasmic membrane (N) | SbcC |
|  | **HA1_08347** | 136 | phage-associated protein-like protein (COG3600) | uncharacterized phage-associated protein [*Butyrivibrio fibrisolvens* 16/4] | 2E-24 | 44% (58/140) | Cytoplasmic (N) | GepA superfamily |
|  | **HA1_08362** | 61 | hypothetical protein | conserved hypothetical protein [*Clostridium perfringens* B str. ATCC 3626] | 2E-27 | 98% (60/61) | Cytoplasmic (N) |  |
|  | **HA1_08367** | 466 | KID repeat-containing protein (COG0419 ATPase involved in DNA repair) | KID repeat family protein [*Clostridium perfringens* B str. ATCC 3626] | 0 | 99% (464/1926) | Extracellular (N) | SMC_prok_B |
|  |  |  |  |  |  |  |  |  |
| Contig_4 | **HA1_02377** | 211 | acetyltransferase (COG0110 acetyltransferase, isoleucine patch superfamily) | acetyltransferase [*Clostridium perfringens* D str. JGS1721] | E-110 | 95% (201/214) | Cytoplasmic (N) | LbH_AT_putative, LbetaH superfamily, NeuD_NnaD |
|  | **HA1_02382** | 385 | capsular polysaccharide biosynthesis protein (COG0438 glycosyltransferase) | capsular polysaccharide biosynthesis protein [*Clostridium perfringens* E str. JGS1987] | 0 | 91% (353/385) | Cytoplasmic (N) | GT1_cap1E_like, Glycosyltransferase_GTB_type superfamily, RfaG |
|  | **HA1_02497** | 136 | acetyltransferase (COG0110 acetyltransferase, isoleucine patch superfamily) | hypothetical protein RTO_07270 [*Ruminococcus torques* L2-14] | 4E-28 | 43% (58/190) | Unknown (N) | LbH_MAT_like, LbetaH superfamily |
|  | **HA1_02502** | 274 | glycosyl transferase family 2 (COG0463 glycosyltransferases involved in cell wall biogenesis) | glycosyl transferase family 2 [*Sebaldella termitidis* ATCC 33386] | 3E-45 | 38% (106/281) | Unknown (N) | Glyco_tranf_GTA_type superfamily, COG1216 |
|  | **HA1_02507** | 286 | glycosyl transferase family 2 (COG0463 glycosyltransferases involved in cell wall biogenesis) | glycosyl transferase family 2 [*Paenibacillus curdlanolyticus* YK9] | 6E-50 | 39% (112/349) | Cytoplasmic membrane (N) | Glyco_tranf_GTA type superfamily |
|  | **HA1_02512** | 398 | hypothetical protein | hypothetical protein bthur0011_49750 [*Bacillus thuringiensis* serovar huazhongensis BGSC 4BD1] | 4E-11 | 26% (64/419) | Cytoplasmic membrane (Y) 0.149 | |
|  | **HA1_02517** | 286 | family 2 glycosyl transferase (COG0463 glycosyltransferases involved in cell wall biogenesis) | glycosyl transferase family 2 [*Lachnospiraceae bacterium* 5_1_63F] | 1E-30 | 32% (84/498) | Cytoplasmic membrane (N) | Glyco_tranf_GTA type superfamily |
|  | **HA1_02522** | 501 | flippase (COG2244 membrane protein involved in the export of O-antigen and teichoic acid) | polysaccharide biosynthesis protein [*Exiguobacterium sibiricum* 255-15] | 3E-68 | 32% (162/511) | Cytoplasmic membrane (Y) 0.135 | |
|  |  |  |  |  |  |  |  |  |
| Contig_6 | **HA1_13597** | 361 | hypothetical protein | No hits found | |  | Unknown (Y) 0.993 |  |
|  | **HA1_13067** | 173 | hypothetical protein (COG1848 predicted nucleic acid-binding protein, contains PIN domain) | No hits found | |  | Unknown (N) | PIN_SF superfamily |
|  | **HA1_13072** | 55 | hypothetical protein | No hits found | |  | Cytoplasmic (N) |  |
|  | **HA1_13077** | 183 | hypothetical protein | membrane-bound pili assembly protein FimC [*Candidatus Pelagibacter ubique* HTCC1062] | 0.001 | 26% (44/239) | Unknown (N) |  |
|  | **HA1_13082** | 150 | hypothetical protein | hypothetical protein [*Plasmodium berghei* strain ANKA] | 0.02 | 34% (25/333) | Cytoplasmic membrane (N) |  |
|  | **HA1_13087** | 120 | hypothetical protein | hypothetical protein ATC3_0055 [*Clostridium perfringens* E str. JGS1987] | 4E-35 | 69% (74/120) | Cytoplasmic (N) |  |
|  | **HA1_13092** | 179 | hypothetical protein | hypothetical protein AC7_2521 [*Clostridium perfringens* NCTC 8239] | 2E-32 | 50% (80/179) | Unknown (N) |  |
|  | **HA1_13097** | 227 | RNA polymerase sigma-F factor (COG0568 DNA-directed RNA polymerase, sigma subunit) | sigma factor [*Clostridium perfringens* D str. JGS1721] | E-116 | 92% (209/232) | Cytoplasmic (N) | Sigma70_r2 superfamily, Sigma70_r4 superfamily, PRK08295 |
|  | **HA1_13102** | 223 | hypothetical protein | hypothetical protein AC5_2412 [*Clostridium perfringens* CPE str. F4969] | E-121 | 94% (211/223) | Cytoplasmic (N) |  |
|  | **HA1_13107** | 170 | hypothetical protein | hypothetical protein AC5_2613 [*Clostridium perfringens* CPE str. F4969] | 3E-83 | 85% (146/170) | Cytoplasmic (N) |  |
|  | **HA1_13112** | 53 | hypothetical protein | conserved domain protein [*Clostridium perfringens* E str. JGS1987] | 1E-08 | 100% (28/46) | Extracellular (N) |  |
|  | **HA1_13117** | 371 | hypothetical protein | RES domain protein [*Butyrivibrio crossotus* DSM 2876] | 2E-66 | 38% (138/363) | Cytoplasmic (N) | RES superfamily |
|  | **HA1_13122** | 308 | hypothetical protein | conserved hypothetical protein [*Clostridium perfringens* E str. JGS1987] | E-117 | 68% (211/308) | Unknown (N) |  |
|  | **HA1_13127** | 281 | hypothetical protein | conserved hypothetical protein [*Clostridium perfringens* E str. JGS1987] | E-147 | 92% (261/281) | Unknown (N) |  |
|  | **HA1_13132** | 179 | hypothetical protein (COG2214 DnaJ-class molecular chaperone) | DnaJ domain protein [*Clostridium perfringens* E str. JGS1987] | 2E-38 | 63% (89/140) | Cytoplasmic (N) | DnaJ superfamily |
|  | **HA1_13137** | 47 | hypothetical protein | hypothetical protein AC3_0048 [*Clostridium perfringens* E str. JGS1987] | 8E-07 | 68% (32/47) | Unknown (N) |  |
|  | **HA1_13142** | 66 | hypothetical protein | hypothetical protein AC5_2617 [*Clostridium perfringens* CPE str. F4969] | 1E-14 | 66% (44/66) | Cytoplasmic (N) |  |
|  | **HA1_13147** | 141 | hypothetical protein | hypothetical protein AC7_2528 [*Clostridium perfringens* NCTC 8239] | 2E-74 | 97% (138/141) | Cytoplasmic (N) |  |
|  | **HA1_13152** | 36 | hypothetical protein | hypothetical protein CJD_A0709 [*Clostridium perfringens* D str. JGS1721] | 3E-11 | 100% (36/39) | Unknown (N) |  |
|  | **HA1_13157** | 433 | replicative DNA helicase (COG0305 replicative DNA helicase) | replicative DNA helicase [*Clostridium perfringens* NCTC 8239] | 0 | 98% (425/433) | Cytoplasmic (N) | DnaB superfamily, P-loop NTPase superfamily |
|  | **HA1_13162** | 76 | hypothetical protein | hypothetical protein AC7_2530 [*Clostridium perfringens* NCTC 8239] | 9E-03 | 55% (22/59) | Unknown (N) |  |
|  | **HA1_13167** | 86 | phage replication initiation protein | phage replication initiation protein [*Clostridium difficile* QCD-76w55] | 1E-15 | 48% (40/243) | Unknown (N) |  |
|  | **HA1_13172** | 213 | hypothetical protein | DNA replication protein DnaD [*Clostridium perfringens* C str. JGS1495] | 5E-48 | 50% (102/296) | Cytoplasmic (N) |  |
|  | **HA1_13177** | 33 | hypothetical protein | No hits found | |  | Extracellular (N) |  |
|  | **HA1_13182** | 74 | hypothetical protein | hypothetical protein AC5_2620 [*Clostridium perfringens* CPE str. F4969] | 9E-35 | 97% (72/74) | Unknown (N) |  |
|  | **HA1_13187** | 101 | hypothetical protein | hypothetical protein phiETA3_gp27 [*Staphylococcus* phage phiETA3] | 5E-07 | 33% (27/101) | Unknown (N) |  |
|  | **HA1_13192** | 69 | hypothetical protein (COG1476 predicted transcriptional regulators) | transcriptional regulator [*Clostridium perfringens* NCTC 8239] | 1E-29 | 100% (69/69) | Unknown (N) | HTH_XRE superfamily |
|  | **HA1_13197** | 170 | DNA-binding protein (COG1396 predicted transcriptional regulators) | transcriptional regulator, Cro/CI family [*Clostridium perfringens* NCTC 8239] | 1E-93 | 100% (170/170) | Cytoplasmic (N) | HTH_XRE superfamily |
|  | **HA1_13202** | 163 | gp23-like protein | phage protein [*Clostridium perfringens* NCTC 8239] | 1E-83 | 100% (151/151) | Cytoplasmic (N) | DUF955 superfamily |
|  | **HA1_13207** | 63 | hypothetical protein | conserved hypothetical protein [*Clostridium perfringens* NCTC 8239] | 2E-17 | 100% (47/53) | Cytoplasmic membrane (Y) 0.888 | |
|  | **HA1_13212** | 400 | phage integrase (COG0582 integrase) | phage integrase [*Clostridium perfringens* NCTC 8239] | 0 | 95% (382/400) | Unknown (N) | phiLC3 phage and phage-related integrases, DNA_BRE_C superfamily, phage_integrase |
|  | **HA1_13597** | 382 | hypothetical protein | putative lipoprotein (*Clostridium perfringens* D str. JGS1987) | 4E-04 | 51% (32/310) | Unknown (Y) 0.993 |  |
|  |  |  |  |  |  |  |  |  |
| Contig_8 | **HA1_07967** | 640 | NADH:flavin oxidoreductase (COG1902 NADH:flavin oxido reductases, Old Yellow Enzyme family) | NADH oxidase [*Clostridium perfringens* D str. JGS1721] | 0 | 99% (637/640) | Cytoplasmic (N) | OYE_like_FMN family, TIM_phosphate_binding superfamily, Pyr_redox superfamily, |
|  | **HA1_07972** | 253 | alpha/beta fold family hydrolase (COG1073 hydrolases of the alpha/beta superfamily) | hydrolases of the alpha/beta superfamily [*Clostridium perfringens* CPE str. F4969] | E-148 | 100% (253/253) | Cytoplasmic (N) | Esterase_lipase superfamily |
|  | **HA1_07977** | 436 | glucuronide permease (COG2211 Na+/melbiose symporter and related transporters) | glucuronide permease [*Clostridium perfringens* E str. JGS1987] | 0 | 95% (418/454) | Cytoplasmic membrane (N) | PRK11462 superfamily, MelB |
|  | **HA1_07982** | 277 | AraC-type sugar metabolism regulator (COG2207 AraC-type DNA-binding domain-containing proteins) | AraC-type sugar metabolism regulator [*Clostridium perfringens* CPE str. F4969] | E-136 | 89% (249/277) | Cytoplasmic (N) | HTH_AraC superfamily |
|  | **HA1_07987** | 435 | hypothetical protein (COG1593 TRAP-type C4-dicarboxylate transport system, large permease component) | C4-dicarboxylate transport system [*Clostridium perfringens* CPE str. F4969] | 0 | 82% (360/435) | Cytoplasmic membrane (Y) 0.425 | ArsB_NhaD_permease superfamily, DctM |
|  | **HA1_07992** | 136 | C4-dicarboxylate transport system permease small protein (COG3090 TRAP-type C4-dicarboxylate transport system, small permease component) | C4-dicarboxylate transport system permease small protein [*Clostridium perfringens* NCTC 8239] | 9E-70 | 100% (136/170) | Cytoplasmic membrane (Y) 0.064 | DctQ superfamily |
|  | **HA1_07997** | 286 | TRAP dicarboxylate transporter, DctP (COG1638 TRAP-type C4-dicarboxylate transport system, periplasmic component) | C4-dicarboxylate transport system substrate-binding protein [*Clostridium perfringens* CPE str. F4969] | E-160 | 99% (285/335) | Cytoplasmic (Y) 0.991 | SBP_bac_7 superfamily |
|  |  |  |  |  |  |  |  |  |
| Contig_9 | **HA1_07162** | 135 | integrase/recombinase (COG0582 integrase) | DNA integration/recombination protein [*Clostridium perfringens* E str. JGS1987] | 2E-61 | 86% (115/152) | Unknown (N) | DNA_BRE_C superfamily |
|  | **HA1_07167** | 82 | hypothetical protein | hypothetical protein AC3_1523 [*Clostridium perfringens* E str. JGS1987] | 1E-39 | 98% (81/82) | Cytoplasmic (N) |  |
|  | **HA1_07172** | 297 | hypothetical protein | hypothetical protein AC3_1524 [*Clostridium perfringens* E str. JGS1987] | E-149 | 99% (266/267) | Cytoplasmic (N) |  |
|  | **HA1_07182** | 93 | DNA-binding protein (COG1396 predicted transcriptional regulators) | DNA-binding protein [*Clostridium perfringens* E str. JGS1987] | 4E-33 | 100% (73/93) | Unknown (N) | HTH_XRE superfamily |
|  | **HA1_07187** | 192 | hypothetical protein | hypothetical protein AC3_1527 [*Clostridium perfringens* E str. JGS1987] | 5E-80 | 80% (155/192) | Unknown (Y) 0.492 |  |
|  | **HA1_07192** | 75 | hypothetical protein | conserved hypothetical protein [*Clostridium perfringens* E str. JGS1987] | 8E-36 | 100% (75/75) | Cytoplasmic (N) |  |
|  | **HA1_07197** | 440 | hypothetical protein (COG4748 uncharacterized conserved protein) | hypothetical protein AC3_1529 [*Clostridium perfringens* E str. JGS1987] | 0 | 92% (406/440) | Cytoplasmic (N) | HSDR_N superfamily |
|  | **HA1_07327** | 397 | hypothetical protein | hypothetical protein CJD_1553 [*Clostridium perfringens* D str. JGS1721] | 0 | 82% (378/459) | Cytoplasmic membrane (Y) 0.623 | Glyco_hydro_42 superfamily |
|  | **HA1_07332** | 486 | hypothetical protein (COG4267 predicted membrane protein) | putative membrane protein [*Clostridium perfringens* D str. JGS1721] | 0 | 86% (422/486) | Cytoplasmic membrane (N) |  |
|  | **HA1_07337** | 469 | hypothetical protein (COG0438 glycosyltransferase) | glycosyltransferase I [*Clostridium perfringens* NCTC 8239] | 0 | 100% (469/469) | Cytoplasmic (N) | GT1_like_3, Glycosyltransferase_GTB_type superfamily, RfaG |
|  | **HA1_07342** | 607 | hypothetical protein (COG4878 uncharacterized protein conserved in bacteria) | conserved hypothetical protein [*Clostridium perfringens* NCTC 8239] | 0 | 95% (581/607) | Cytoplasmic membrane (Y) 0.287 | COG4878 superfamily, DUF2194 |
|  | **HA1_07347** | 286 | hypothetical protein | conserved hypothetical protein [*Clostridium perfringens* B str. ATCC 3626] | E-140 | 92% (264/298) | Cytoplasmic (N) |  |
|  | **HA1_07352** | 679 | hypothetical protein | putative fusion: nucleoside-diphosphate-sugar epimerase and gaf domain [*Clostridium perfringens* NCTC 8239] | 0 | 91% (621/679) | Cytoplasmic (N) | WcaG, PRK04023 |
|  |  |  |  |  |  |  |  |  |
| contig 00062 | **HA1_06482** | 72 | hypothetical protein | two-component sensor histidine kinase (*Candidatus Pelagibacter ubique* HTCC1002) | 1.7 | 44% (32/512) | Cytoplasmic (N) |  |
|  | **HA1_06487** | 61 | hypothetical protein | conserved hypothetical protein (*Clostridium perfringens* B str. ATCC 3626) | 2E-18 | 80% (47/60) | Cytoplasmic (N) |  |
|  | HA1_06492 | 259 | DNA adenine methylase (Dam) | putative modification methylase dpniia (*Clostridium* phage phiSM101) | 2E-138 | 93% (241/259) | Unknown (N) | Site-specific DNA methylase |
|  | **HA1_06497** | 377 | putative N-acetylmuramoyl-L-alanine amidase (uncharacterized protein involved in peptidoglycan biosynthesis) | N-acetyl-L-alanine amidase domain protein (*Clostridium perfringens* D str. JGS1721) | 4E-88 | 72% (162/415) | Cytoplasmic (N) | Peptidoglycan recognition proteins (PGRPs), Bacteria SH3 domain |
|  | **HA1_06502** | 301 | hypothetical protein | hypothetical protein CBC_A1709 (*Clostridium botulinum* C str. Eklund) | 7E-12 | 24% (70/312) | Cytoplasmic (N) |  |
|  | **HA1_06507** | 200 | hypothetical protein | hypothetical protein HMPREF9384_0503 (*Streptococcus sanguinis*) | 1E-06 | 27% (54/191) | Cytoplasmic membrane (N) |  |
|  | **HA1_06512** | 141 | hypothetical protein | acyl-CoA dehydrogenase domain-containing protein (*Stenotrophomonas maltophilia* R551-3) | 4.1 | 31% (18/545) | Extracellular (N) |  |
