# Supplementary material for: Genome Sequencing and Analysis of a Type A Clostridium perfringens Isolate from a Case of Bovine Clostridial Abomasitis
Source: PLoS One. 2012 Mar 8;7(3):e32271. doi: 10.1371/journal.pone.0032271 (PMC3297601; doi:10.1371/journal.pone.0032271)
Supplement: Table S4 — PanSeq results for the orphan contigs. (DOC) [file pone.0032271.s005.doc]

Table S4: PanSeq results for the orphan contigs

The orphan contigs were compared against the *C. perfringens* Strain 13, ATCC 13124 and SM101 chromosomes using PanSeq and the unique regions identified. Regions that remained unique after comparison to all complete *C. perfringens* plasmid and phage sequences are bolded. Everything remained unique in c85c46 and contig00028. Nothing remained unique in contig00020 and there were mixed results in c92ic19.

| **Contig** | **Locus_tag** | **Size (aa)** | **Predicted Product (notes in brackets)** | **Hit Description** | **E-value** | **% Identity (aa)** | **Subcellular localization** | **Conserved domains** |
| --- | --- | --- | --- | --- | --- | --- | --- | --- |
|  |  |  |  |  |  |  |  |  |
| contig00020 (1-2262/2262) | HA1_16112 | 62 | hypothetical protein | hypothetical protein AC1_A0171 (*Clostridium perfringens* B str. ATCC 3626) | 1E-17 | 76% (47/71) | Cytoplasmic membrane (Y) 0.103 |  |
|  | HA1_16117 | 97 | hypothetical protein | transcriptional regulator, MerR family (*Subdoligranulum variabile* DSM 15176) | 0.09 | 37% (17/298) | Unknown (N) |  |
|  | HA1_16122 | 189 | resolvase (site-specific recombinases, DNA invertase Pin homologs) | resolvase (*Clostridium perfringens* B str. ATCC 3626) | 2E-100 | 98% (185/190 ) | Unknown (N) | Serine recombinase (SR) family, resolvase and invertase subfamily, catalytic domain |
|  | HA1_16127 | 58 | hypothetical protein (uncharacterized conserved protein) | hypothetical protein (*Pectobacterium atrosepticum*) | 9E-15 | 79% (37/51) | Unknown (N) | Domain of unknown function |
|  |  |  |  |  |  |  |  |  |
| c85c46 (1-2586/2586) | **HA1_15927** | 374 | hypothetical protein | hypothetical protein GCWU000182_00922 (*Abiotrophia defectiva* ATCC 49176) | 1E-31 | 30% (109/560 ) | Cytoplasmic (N) |  |
|  | **HA1_15915** | 128 | transglutaminase/protease (uncharacterized protein involved in cytokinesis) | hypothetical protein GCWU000182_00922 (*Abiotrophia defectiva* ATCC 49175) | 5E-18 | 42% (53/560) | Unknown (N) | Transglutaminase/protease-like homologs |
|  | **HA1_15920** | 50 | hypothetical protein | No significant similarity found |  |  | Cell wall (N) |  |
|  |  |  |  |  |  |  |  |  |
| c92ic19 (1-4659/10216) | **HA1_15932** | 245 | hypothetical protein | hypothetical protein CPC_A0084 (*Clostridium perfringens* C str. JGS1495) | 7E-131 | 99% (242/246 ) | Cytoplasmic (N) | Telomeric repeat-binding factor 2 |
|  | **HA1_15937** | 362 | hypothetical protein | protein rlx (*Clostridium perfringens* C str. JGS1495) | 0 | 99% (359/362 ) | Cytoplasmic (N) | Relaxase/Mobilisation nuclease domain |
|  | **HA1_15942** | 105 | mobilisation protein | MobC, Bacterial mobilisation protein (*Clostridium perfringens* B str. ATCC 3626) | 2E-47 | 100% (105/105 ) | Unknown (N) |  |
|  | HA1_15947 | 101 | ribbon-helix-helix protein domain-containing protein | ribbon-helix-helix protein, CopG family domain protein (*Clostridium perfringens* C str. JGS1495) | 2E-46 | 97% (98/101) | Unknown (N) |  |
|  | **HA1_15952** | 64 | hypothetical protein | conserved hypothetical protein (*Clostridium perfringens* B str. ATCC 3626) | 4E-30 | 100% (64/64) | Cytoplasmic (N) |  |
|  | **HA1_15957** | 65 | hypothetical protein | bacteriocin-type signal sequence domain protein (*Clostridium perfringens* E str. JGS1987) | 1E-28 | 100% (65/65) | Extracellular (Y) 0.009 |  |
|  | **HA1_15962** | 119 | hypothetical protein | conserved hypothetical protein (*Clostridium perfringens* E str. JGS1987) | 4E-63 | 99% (118/119 ) | Unknown (N) |  |
|  | HA1_15967 | 42 | hypothetical protein | ribbon-helix-helix protein, CopG family domain protein (*Clostridium perfringens* C str. JGS1495) | 1E-11 | 95% (39/101) | Unknown (N) |  |
|  |  |  |  |  |  |  |  |  |
| contig00028 (1-2182/2182) | **HA1_16132** | 639 | sensory box histidine kinase | sensory box histidine kinase (*Clostridium perfringens* D str. JGS1721) | 0 | 99% (636/639) | Cytoplasmic membrane (Y) 0.224 | Histidine kinase-like ATPases |
